# Integrative modeling reveals the principles of multi-scale chromatin boundary formation in human nuclear organization

**DOI:** 10.1186/s13059-015-0661-x

**Published:** 2015-05-27

**Authors:** Benjamin L Moore, Stuart Aitken, Colin A Semple

**Affiliations:** MRC Human Genetics Unit, MRC Institute of Genetics and Molecular Medicine, University of Edinburgh, Crewe Road, Edinburgh, EH4 2XU UK

## Abstract

**Background:**

Interphase chromosomes adopt a hierarchical structure, and recent data have characterized their chromatin organization at very different scales, from sub-genic regions associated with DNA-binding proteins at the order of tens or hundreds of bases, through larger regions with active or repressed chromatin states, up to multi-megabase-scale domains associated with nuclear positioning, replication timing and other qualities. However, we have lacked detailed, quantitative models to understand the interactions between these different strata.

**Results:**

Here we collate large collections of matched locus-level chromatin features and Hi-C interaction data, representing higher-order organization, across three human cell types. We use quantitative modeling approaches to assess whether locus-level features are sufficient to explain higher-order structure, and identify the most influential underlying features. We identify structurally variable domains between cell types and examine the underlying features to discover a general association with cell-type-specific enhancer activity. We also identify the most prominent features marking the boundaries of two types of higher-order domains at different scales: topologically associating domains and nuclear compartments. We find parallel enrichments of particular chromatin features for both types, including features associated with active promoters and the architectural proteins CTCF and YY1.

**Conclusions:**

We show that integrative modeling of large chromatin dataset collections using random forests can generate useful insights into chromosome structure. The models produced recapitulate known biological features of the cell types involved, allow exploration of the antecedents of higher-order structures and generate testable hypotheses for further experimental studies.

**Electronic supplementary material:**

The online version of this article (doi:10.1186/s13059-015-0661-x) contains supplementary material, which is available to authorized users.

## Background

The chromatin structure of human interphase chromosomes plays critical roles in a wide range of cellular functions and consists of many hierarchically arranged but interconnected layers of structure. These range from the three-dimensional arrangement of multi-megabase-scale domains within the nucleus down to the chemical modifications carried by individual nucleosomes and nucleotides at particular loci. A recurring question has been how these many different levels of chromatin structure are related to one another [1]. In the wake of recent efforts to comprehensively map the epigenomic landscape in human cells, integrative approaches have suggested classifications of chromatin into distinct, functional states. The number of chromatin states identified in these pioneering studies has varied widely, from as few as 6 to as many as 51, using a variety of locus-level features such as DNA methylation, histone modifications and transcription factor binding patterns [2-5]. These states usually encompass small, sub-genic regions and have provided intriguing insights into chromatin-mediated variation in promoter and enhancer activity. At the same time technological developments such as the Hi-C method have provided datasets describing the overall spatial organization of the human genome [6], but the relationships between such datasets and the wide spectrum of locus-level features are not well understood. A recent study examining seven such features and their relationships to the spatial organization of the mouse genome in embryonic stem cells (ESCs) concluded that chromosome architecture is largely determined by the binding patterns of particular transcription factors, and that these cells have a unique higher-order chromatin structure as a result [7]. Thus it is unclear whether such results are relevant to other cell types and species, or whether the inclusion of a broader range of features would provide additional insights.

Many aspects of higher-order chromatin remain broadly invariant between cell types, and genome-wide datasets as diverse as replication timing domains, lamin association domains and Hi-C interaction matrix eigenvectors show strong correlations across many different human cell lines [8]. Indeed, most measurable aspects of higher-order structure have been conserved during evolution across the majority of the mammalian genome [8-10]. However, a minority (perhaps 20% to 30%) of the genome is within more labile structures, such that the behaviors of many replication timing domains and lamin association domains change significantly upon cellular differentiation from ESCs, altering the transcriptional output of many resident genes [10,11]. A large literature surrounds the dynamics of locus-level chromatin during differentiation and reprogramming, emphasizing the critical importance of genomic patterns of DNA binding proteins, particular histone modifications and DNA methylation (for example, [12]). Yet we still lack an integrated view of chromatin dynamics that details the dependencies between these locus-level phenomena, the remodeling of large domains and changes in nuclear organization. The extent to which higher-order chromatin dynamics depends upon the spectra of features occurring at these lower levels has not been studied quantitatively.

Given the existence of neighboring chromatin domains with distinct structures and activities, the boundaries defining such domains have been a focus of particular interest. The topological domains (TADs) described by Dixon et al. [9] were reported to be separated by boundary regions showing pronounced peaks of the insulator binding protein CTCF, although depletion of CTCF appears to have little effect on TAD boundaries [13]. Similarly, deletion of a TAD boundary on the mouse X chromosome resulted in many altered interactions, but did not cause the two TADs separated by this boundary to completely merge [14]. Thus there is much left to learn about the basis of TAD boundaries. The scale of TAD organization (median length 880 kb) is below that of the multi-megabase chromatin domains delineating occupancy of A and B nuclear compartments [15]. These compartments constitute domains of transcriptionally active, relatively centrally positioned chromatin, and relatively inactive, peripheral chromatin respectively; consequently compartment boundaries often mark a profound divergence in functional state. It is not known whether TAD boundaries coincide with compartment boundaries, and the similarities or differences in the features underlying these two boundary classes also remain unstudied.

Here we exploit the unprecedented volumes of data produced recently [4] to provide an integrated and rigorously quantitative view of locus-level chromatin features, higher-order chromatin structure and nuclear organization across three cell types. We use integrative modeling approaches to directly study the contribution of 35 locus-level chromatin features to chromosome architecture across three human cell types as measured by Hi-C. These data are relevant to the quantitative, molecular basis of higher-order chromatin, the dominant determinants of chromatin dynamics, and prominent features conferring the structure of domain boundaries.

## Results

### Higher-order chromatin organization is largely concordant and predictable across cell types

**Figure 1:**
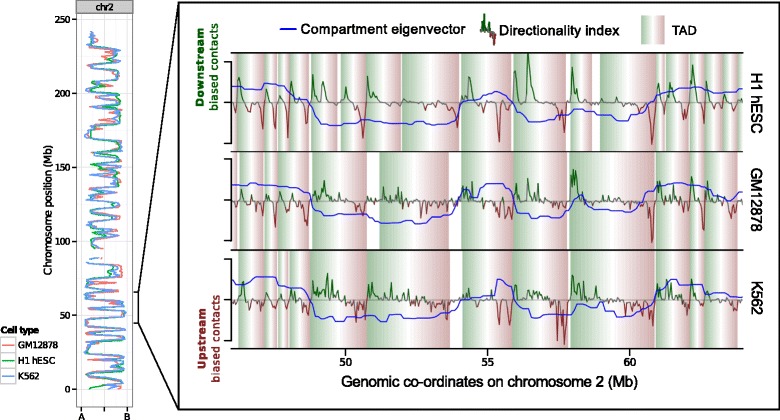
Concordance of chromatin structure at multiple scales over three human cell types. The eigenvector compartment profile is shown for chromosome 2 for three human cell types (left). Genome-wide Pearson correlation coefficients between eigenvectors at 1-Mb resolution are in the range 0.75 to 0.80 (Additional file 1: Figure S2B). At higher resolution, the zoomed region illustrates conservation of topological domains (TADs) over 20 Mb of the same chromosome. Genome-wide, 33% of all H1 TAD boundaries have a matching boundary in GM12878 in the same or an adjacent 40-kb bin (K562: 31%, null model: 18%; Kolmogorov–Smirnov test: *D*=0.26, *p*≈0). Similarly, for H1 compartment boundaries, 37% have a matching boundary in the same or an adjacent 100-kb bin in GM12878 (K562: 35%, null model: 7%; Kolmogorov–Smirnov test: *D*=0.47, *p*≈0, Additional file 1: Figure S2A). Mb, megabases; TAD, topological domain.

In common with previous studies of higher-order chromatin structure [8-11], there was evidence for good concordance of Hi-C data between different cell types. Hi-C eigenvectors were calculated for three human cell types (GM12878, H1 hESC and K562 cell lines) using the same analysis protocols, and were found to be strongly and significantly correlated (Figure 1; Additional file 1: Figure S1). Most 1-Mb regions appear to be constitutively present (that is, across cell types) in either the A or B compartments, corresponding to relatively centrally positioned, transcriptionally active or more peripheral repressive chromatin, respectively [15]. Strong correspondence across cell types was also observed for TAD boundaries, and for the positioning of compartment boundaries, separating A and B compartments (Additional file 1: Figure S2).

**Figure 2:**
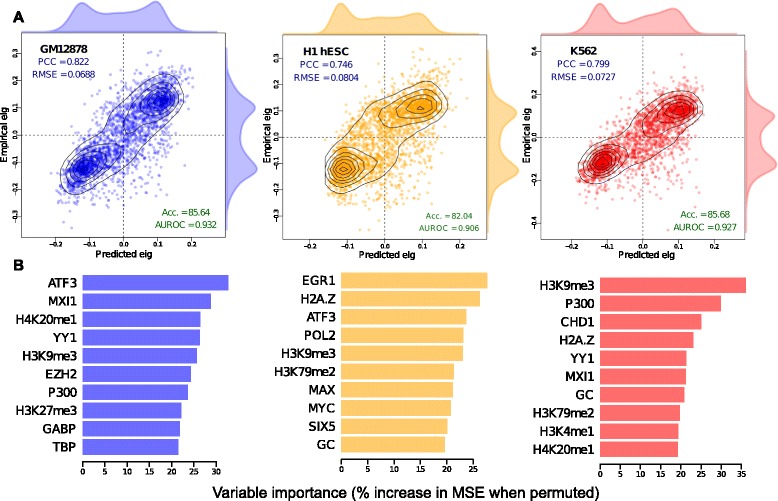
Accurate models of higher-order chromatin state built from locus-level features.**(A)** Model predictions (Predicted eig) are compared to observed values (Empirical eig). Various metrics are used to measure the accuracy of regression modeling – the Pearson correlation coefficient (PCC) and root mean-squared error (RMSE) – and to evaluate classification accuracy (for A *e* *i* *g*≥0 and B *e* *i* *g*<0) – accuracy (percentage of true positives, Acc.) and area under the receiver operating characteristic curve (AUROC). **(B)** Variable importance (shown for the ten most informative features per model) is calculated as the decrease in the accuracy of predictions for the permuted variable relative to observed (in units of percentage increase in MSE), averaged over the forest (see Materials and methods). Acc., accuracy; AUROC, area under the receiver operating characteristic curve; eig, eigenvector; MSE, mean-squared error; PCC, Pearson correlation coefficient; RMSE, root mean-squared error.

Although it is often assumed that higher-order chromatin domain organization (at the megabase scale) across the genome is to some degree dependent upon lower-level features (at the scale of tens or hundreds of base pairs), the identity and independent contributions of these features are unknown. Beyond this it has also been unclear whether there are strong enough dependencies to allow accurate prediction of higher-order structure. For each of the three Hi-C eigenvector datasets corresponding to the Tier 1 ENCODE cell lines (GM12878, H1 hESC and K562) we assembled datasets of 35 matched locus-level chromatin features, including sites bound by 21 DNA binding proteins, and 11 histone modifications/variants and DNase hypersensitive sites (see Materials and methods). The GC content of each 1-Mb region, which is known to be correlated with higher-order structure (for example, [8]), was also included as an additional feature in each model for comparison with chromatin features. Importantly, each Hi-C dataset was re-analyzed to provide comparable identically processed data, which was complementary to the identically processed, locus-level ENCODE data. It was possible to construct random forest models with good predictive accuracy, and strong and significant correlations were seen between predicted and empirically measured eigenvector values for each cell type (Figure 2). The models show high predictive power, particularly for GM12878 where the model achieved a Pearson correlation coefficient (PCC) of 0.805 between predicted and measured values. These levels of accuracy are similar to those reported (median PCC =0.83 over seven cell types) for strikingly successful models of the transcriptional output of promoters using locus-level chromatin features [16]. Other evaluation metrics also suggested successful models, such as the ability to correctly assign 1-Mb regions to compartments A and B (see area under the receiver operating characteristic data in Figure 2). It would be feasible to construct similar, but more comprehensive models using all ENCODE chromatin features for a given cell type, although the resulting models would not be comparable between cell types. However, the high accuracy of the current models suggests there is limited potential for improvement by adding further features. Also, even the most comprehensive models that could be constructed, using all currently available data, inevitably represent a minority of the features actually present in chromatin [1].

While 1-Mb compartment eigenvectors are low resolution relative to that typically employed for chromatin immunoprecipitation sequencing (ChIP-seq) data, megabase bins are a suitable choice for analyzing large chromosomal compartments [15,17]. To confirm our modeling accuracy is not sensitive to resolution, we applied models trained with 1 Mb to 100 kb resolution datasets and saw similarly high levels of accuracy (88% to 95 *%*, as accurate as 1-Mb models in terms of predicted and empirical PCC, Additional file 1: Figure S3).

### Influential features underlying higher-order structure differ between cell types

Given the correlations seen between Hi-C eigenvectors from different cell types (Figure 1) and the similar predictive power of cell-type-specific models (Figure 2A), one might assume that a similar combination of informative variables appears in each of the models. The broad trends in relative variable importance (see Materials and methods) do indeed suggest that many features have a similar influence in each of the three models (Additional file 1: Figure S4A). For example the genomic distributions of CTCF binding patterns, H3K36me3, H3K27ac and GC content maintain very similar influence across all three models, while certain variables depart from this trend and show a notably higher variable importance in a particular model. Thus substantial levels of variation between cell types are seen for the top ten most influential variables across models (Figure 2B), such that the repressive histone modification H3K9me3 is the only feature, among the ten most influential, shared between all three cell-type models. This is expected since H3k9me3 is anticorrelated or uncorrelated with most other input features (Additional file 1: Figure S5), and is therefore a relatively information-rich variable. Overall, more highly ranked features are shared between the two relatively differentiated, hematopoietic cell lines (GM12878 and K562), with the pluripotent ESC line (H1 hESC) showing more distinct characteristics. The EGR1 transcription factor plays critical roles in cellular differentiation and shows markedly higher variable importance in the H1 hESC model. While the P300 transcriptional co-activator protein, which controls the proliferation and differentiation of hematopoietic progenitor cells, ranks more highly in the two hematopoietic cell line models (Figure 2B, Additional file 1: Figure S4).

Many of the variables examined here are heavily interdependent, and for example co-occur in clusters denoting functional chromatin states [4]. Care must be taken not to over-interpret the differences in variable importance between models, given the pervasive multi-collinearity and clustering between variables in the input locus-level feature set (Additional file 1: Figure S5). For instance, MXI1 is an influential feature in both the hematopoietic models, while MYC and MAX are among the highest ranked features in the H1 hESC model. This is in keeping with recent results suggesting MYC binds open chromatin as a transcriptional amplifier in ESCs [18,19], with MAX and MXI1 long being known as antagonistic co-regulators of MYC [20]. Thus, in identifying nominally different informative variables for each model we will, to some extent, select different representatives of the same cluster (Additional file 1: Figure S5). It follows that we would expect a large number of different feature combinations to have similar predictive power in broadly equivalent random forest models. With a broader perspective, there are general similarities across all three models, in that all derive much of their predictive power from indicators of transcriptional activity, markers of heterochromatin and the binding levels of combinations of broadly expressed transcription factors (Additional file 1: Figure S6).

**Figure 3:**
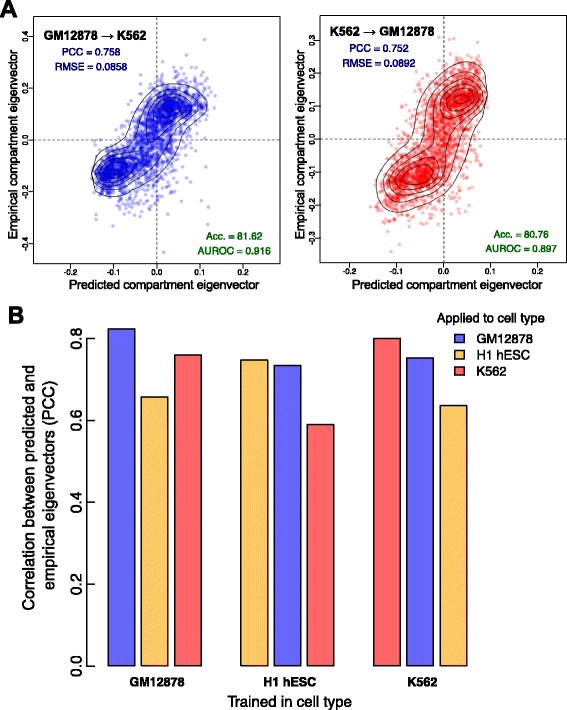
Models trained in one cell type can generalize to others. Each model, trained in one cell type, was applied to the chromatin feature datasets from the other two cell types. **(A)** The GM12878 model achieved high accuracy when applied to K562 features (PCC =0.76), as did the reciprocal cross (PCC =0.75). **(B)** In each case, predictive accuracy decreased on cross-application but there remains significant agreement between predicted and empirical values. Acc., accuracy; AUROC, area under the receiver operating characteristic curve; PCC, Pearson correlation coefficient; RMSE, root mean-squared error.

Consistent with the presence of broad commonalities among the three models, cross-application of models showed that models trained in one cell type often performed well in another (Figure 3). In each instance of cross-application, predictive accuracy declined by no more than 21% relative to the model’s native cell type. In reciprocal crosses between the two hematopoietic cell lines (K562 and GM12878), this loss of accuracy was between 5.9% and 7.8% (Figure 3A), but was 20.2% to 20.4% when these models were applied to H1 hESC data. This again highlights the relatively unusual structural features of the pluripotent state.

We compared the performance of our random forest approach with two other regression methods: simple multiple linear regression and partial least squares regression, a method particularly well suited to highly correlated inputs [21]. While cell-type-specific prediction accuracy remained high for each method, cross-application between cell types confirmed our random forest approach as that most capable of learning generalizable rules of compartment prediction (Additional file 1: Figure S7).

### Regions of variable structure are enriched for cell-type-specific enhancers

**Figure 4:**
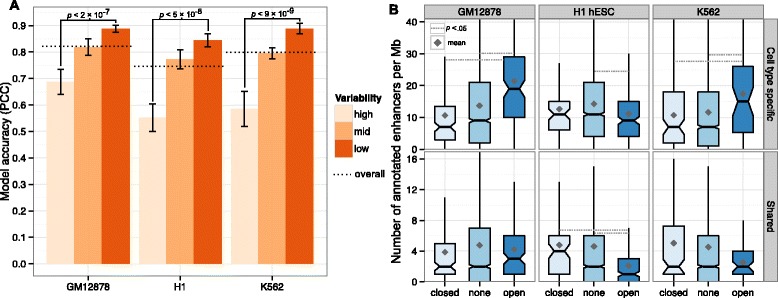
Regions with variable structure are less successfully modeled and are associated with cell-type-specific enhancer activity.**(A)** Model accuracy is significantly different between low- and high-variability regions, defined as, respectively, the lowest and highest thirds of the distribution of median absolute deviations between cell types. **(B)** Regions occupying altered compartments are defined as those assigned to A in one cell type but to B in the other two cell types, or vice versa. The numbers of enhancers (cell type specific or shared between two or more cell types) are shown for regions with altered (open or closed) and non-altered (none) compartments in each cell type. PCC, Pearson correlation coefficient.

Although the chromatin organization of much of the genome appears to be invariant between cell types (Figure 1), some regions are more dynamic. There is a clear relationship between modeling accuracy and structural stability between cell types such that the structures of more variable regions are more challenging to predict. This is evident even with the most liberal definitions of variability; for instance, if we calculate the median absolute deviation between eigenvectors across all three cell types and simply trisect the distribution, we found that the most structurally variable regions between cell types were significantly less accurately modeled in each case (Figure 4A). This could indicate the cell-type-specific features responsible for organizing these regions are largely missing from our training set, which undoubtedly represents a tiny minority of all the actual components of chromatin in real human cells. However, it is unclear whether structural variability defined so broadly reflects altered biological function or is dominated by stochastic variations in structure among cells [22].

A more conservative definition of structurally variable regions is that they are regions altering their compartment state (between A and B compartments) in one cell type relative to the other two. Such regions will often undergo dramatic changes between transcriptionally permissive and repressive environments and might be expected to be associated with cell-type-specific biology, such as functional chromatin states [4]. This indeed seems to be the case, with regions occupying altered compartments showing corresponding changes in enhancer activity. Regions undergoing a B to A compartment transition, to a relatively transcriptionally permissive structure, were enriched for cell-type-specific enhancers in the two derived cell types used in this study but not in the ESC line, which would not be expected to have lineage-specific enhancer contacts active in its pluripotent state (Figure 4B). The same pattern was not seen for enhancers shared between two or more of the cell types under study. We observed a similar enrichment for cell-type-specific transcription (Additional file 1: Figure S8) but not for several other chromatin states including promoter activity (Additional file 1: Figure S9).

**Figure 5:**
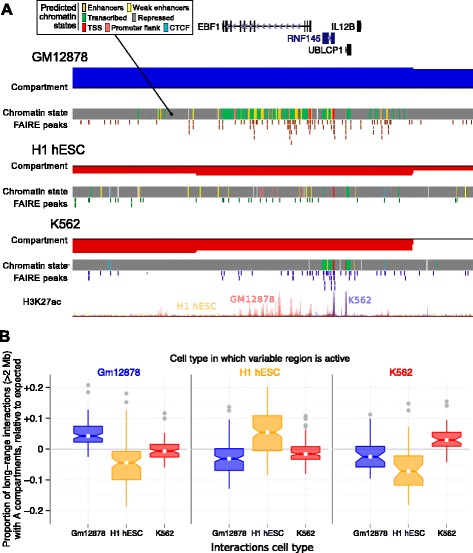
Structurally variable regions indicate cell-type-specific biology. Regions occupying the active A nuclear compartment in one cell type, but the repressed B compartment in the other two, were selected and ranked by the number of predicted active enhancers (Figure 4). **(A)** The region chr5:158–159 Mb, which occupies the open A compartment in GM12878 cells, is shown as an example (top five regions for each cell type are shown in Additional file 1: Figure S10). Displayed tracks are: known genes (UCSC), compartment eigenvectors, chromHMM/Segway combined chromatin state predictions, open chromatin FAIRE peaks, and H3K27ac signal. **(B)** Structurally variable regions show a greater than expected proportion of contacts with other active A compartments, in the cell type in which they are active relative to those same regions in the other two cell types. Box plot notches represent 95 *%* confidence intervals of the median. Each variable region is also shown individually in Additional file 1: Figure S11. TSS, transcription start site.

For each cell line, we identified all regions showing cell-type-specific occupancy of the active A compartment and ranked these regions according to the density of predicted active enhancers. Close examination of these regions reveals many examples of enhancer activity nucleated upon genes associated with cell-type-specific biology (Figure 5A, Additional file 1: Figure S10). For the GM12878 (B-cell derived) cell line, an active region of variable structure rich in active enhancers was found to contain the EBF1 (early B-cell factor 1) gene (Figure 5A). The transcription factor encoded by this gene has been identified as essential in maintaining B-cell identity and establishing early lineage commitment [23,24]. Similarly a variable region active in H1 hESC (Additional file 1: Figure S10B.1) harbors the PAX1 regulator of patterning during embryogenesis [25], while a K562-specific active region (Additional file 1: Figure S10C.3) contains a gene encoding a regulator of hematopoiesis (ZFPM2/FOG2 [26]). Each example is concordant with the known biology of the cell type concerned, and each is illustrative of the genome-wide relationship between higher-order structural variability and cell-type-specific enhancer activity (Figure 4B). We explored the functional annotations of genes in regions of cell-type-specific structure (Additional file 2: Tables S1, S2 and S3), and although we observed some artifactual enrichments (generated by duplicated gene clusters within some of these 1-Mb regions), no significant enrichments were seen across regions.

A defining characteristic of active A compartment regions is a preferential bias in contacting other A compartment regions [15]. However, it is not clear whether cell-type-specific transitions in higher-order structure are solely compartment-level phenomena, or involve other structural strata. We therefore examined the genome-wide contact profiles of each region of variable cell-type-specific chromatin structure in detail. If these cell-type-specific structures are mediated by finer-scale structural levels (such as TADs) we might expect to see predominantly short-range contacts in their underlying contact profile. Instead, we found that variable regions preferentially interact with other A compartment regions in the cell types in which they are active (Figure 5B, Additional file 1: Figure S11), but not in the other cell types in which they are inactive. This supports the idea that these cell-type-specific regions are undergoing compartment-level transitions, disproportionately mediated by the formation of long-range contacts, while also not precluding additional changes at lower levels such as TADs.

### TAD boundaries and compartment boundaries possess similar features

The mammalian genome is organized into TADs, predominantly self-interacting chromatin domains, with boundary regions reportedly associated with pronounced peaks and troughs of particular features within 500 kb of the predicted boundary [9]. Exploration of this phenomenon using a set of 24 mouse ESC chromatin features (and a smaller number of human ESC features) reportedly revealed enrichment peaks of CTCF, H3K4me3 and H3K36me3, as well as a pronounced dip in H3K9me3, suggesting that high levels of transcription may contribute to boundary formation [9]. However, it was unclear whether other features show unusual patterns in TAD boundary regions, and whether the constellation of features involved changes between cell types. The features associated with boundaries separating A and B compartments calculated from Hi-C eigenvectors have not been studied to our knowledge. The datasets assembled here, consisting of 35 matched chromatin features across three cell types, allow us to conduct the first comparative study of the constituents of human TAD and compartment boundary regions.

**Figure 6:**
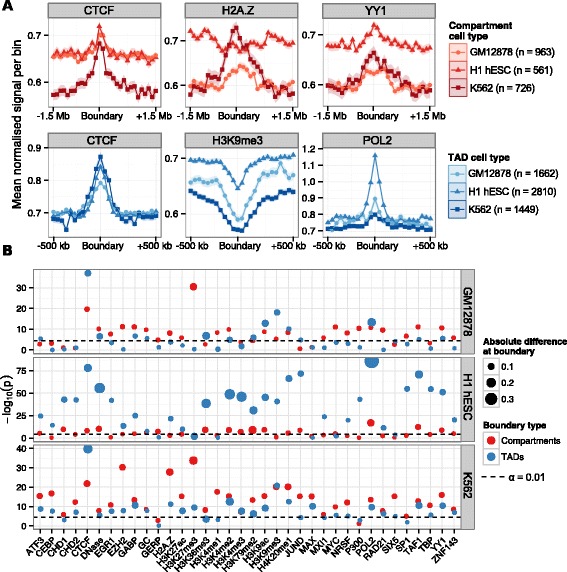
Chromatin features underlying TAD and compartment boundaries.**(A)** Selected profiles for locus-level features are shown for TAD boundaries (CTCF, H3K9me3 and POL2) and compartment boundaries (H2A.Z, H3K4me2 and YY1), as a mean normalized ChIP-seq signal relative to input chromatin per bin (±1 standard error). TAD boundaries were examined over 40-kb bins over the 1 Mb flanking each boundary; compartment boundaries were examined over 100-kb bins over 3 Mb. **(B)** The significance of enrichment or depletion (− log10*P* two-tailed Mann–Whitney test) of a feature was calculated as the boundary bin relative to the ten most peripheral bins (five either side). Points are scaled by the absolute mean difference in signal over the boundary relative to the mean of peripheral bins. ChIP-seq, chromatin immunoprecipitation sequencing; TAD, topological domain.

We derived TAD boundaries according to established methods [9] for all three cell types under study. We then sought evidence for significantly enriched or depleted features at TAD boundary regions using a conservative approach (a nonparametric statistical test and Bonferroni multiple testing correction, see Materials and methods), and confirmed the previously reported peaks (CTCF and POL2) and dip (H3K9me3) in ESC data, but also revealed substantial heterogeneity between cell types. CTCF binding was found enriched at TAD boundaries across all cell types, but other features, including H3K36me3 and H3K4me3, show dramatic peaks of enrichment in H1 hESC cells that are not seen consistently in other cell types (Figure 6, Additional file 1: Figure S12). Although the dip in H3K9me3 at TAD boundaries is seen in all cell types, the extent of the depletion varies and is weakest in H1 hESC cells. Many other features show significant, though often modest, enrichments in a particular cell type. However, overall the complexity of TAD boundaries (measured as the number of strongly enriched features) is notably higher in H1 hESC than in the other two, more differentiated, cell types (Figure 6), involving large increases in the binding of sequence specific factors such as SP1 and JUND.

Across all three cell types, several features demonstrate consistent and statistically significant patterns at TAD boundaries (Figure 6, Additional file 1: Figure S12), including peaks associated with active transcription of genes (POL2 and H3K9ac) and dips in H3K9me3, as previously reported [9]. However, other novel feature peaks of interest emerge across cell types, such as peaks of H4K20me1, a modification previously implicated in chromatin compaction [27]. Significant peaks in YY1 are evident in all cell types, which is intriguing given the evidence that YY1 and CTCF cooperate to affect long-distance interactions [28]. Co-binding of CTCF with YY1 has also been shown to identify a subset of highly conserved CTCF sites [29]. Co-binding of CTCF and YY1 may also therefore be a contributing factor in the establishment of TAD boundaries, which appear to be broadly conserved across mammals [9]. To test this, we split our sets of TAD boundaries into those possessing ChiP-seq peaks (region peaks called by ENCODE [4]) for CTCF, YY1, both CTCF and YY1 (overlapping peaks) and neither. We then tested each boundary subset for genome-wide enrichments of the other features in our dataset (Additional file 1: Figure S14). Unexpectedly, we found that boundaries marked by YY1 (without overlapping CTCF peaks) were generally most strongly enriched for other features in our dataset. We also found that boundaries lacking both CTCF and YY1 peaks showed instead the strongest enrichments for RAD21 in each cell type (Additional file 1: Figure S14), reinforcing previous findings that describe the distinct influences of CTCF and cohesin in organizing chromatin structure [13,30,31]. We also observe consistent increases in GC content at TAD boundaries, at a scale that is difficult to reconcile with the presence of smaller-scale features such as repeat elements or CpG islands (Additional file 1: Figure S12).

Where neighboring genomic regions occupy contrasting A and B nuclear compartments, the disparity implies the presence of a boundary region. Putative compartment boundaries were identified by using a hidden Markov model to infer the state sequence of A/B compartments across the genome based on observed principal component eigenvectors. Analogously to the TAD boundary analysis, we then sought significant enrichments or depletions in 36 chromatin features over these compartment boundaries (Figure 6, Additional file 1: Figure S13). Compartment boundaries display similar spectra of enrichments to previously studied TAD boundaries [9] but at lower resolution, reflecting the different scales of these levels of organization (Figure 6B, Additional file 1: Figure S13). Peaks associated with active promoters (POL2, TAF1 and H3K9ac) are again evident. Parallel enrichments of CTCF, YY1 and H4K20me1 are also seen at compartment boundaries, as they were for TAD boundaries, in each cell type under study. In addition, compartment boundaries show enrichments of H3K79me2, which is known to play critical roles in cellular reprogramming [32]. Remarkably, H3K79me2 has also recently been shown to mark the borders of small regions of open chromatin (hundreds of base pairs) [33]. Thus, there may be similarities in chromatin compaction boundaries at very different scales.

Certain features show intriguing contrasts between cell types. The histone variant H2A.Z lacks any trace of enrichment at H1 hESC compartment boundaries, but is significantly enriched in the other two cell types (Figure 6A), consistent with reports describing H2A.Z relocation during cellular differentiation [34]. Compartment boundaries also show enrichment for the cohesin complex subunit RAD21 in the two hematopoietic cell types (Additional file 1: Figure S12), and cohesin is another factor implicated in modulating nuclear architecture in partnership with CTCF [13]. Various other enrichments with very modest effect sizes are also evident at compartment boundaries (Figure 6B, Additional file 1: Figure S13). In contrast to TAD boundaries, the composition of compartment boundaries appears least complex in H1 hESC, relative to the other two cell types. Overall compartment and TAD boundaries are associated with overlapping spectra of chromatin features across cell types. These involve DNA-binding proteins implicated in chromosome architecture (CTCF, YY1 and RAD21), but also implicate the initiation and repression of transcription as critical to boundary formation. However, these two boundary classes occur at different scales, with patterns of informative features typically spanning regions up to 500 kb for TAD boundaries, and patterns associated with compartment boundaries often spanning more than 1 Mb (Additional file 1: Figure S12, Additional file 1: Figure S13).

### Topological domains cluster by epigenetic enrichments

Sexton et al. [35] showed that, in the *Drosophila* genome, topological structures termed physical domains could observably be clustered into distinct functional groups based on their average feature enrichments. It is of interest to repeat this experiment with our human datasets and across multiple cell types to detect finer delineation of chromatin state beyond A and B compartmentalization. We found that TADs called across the three cell types used in this work could be clustered into transcriptionally active (active), repressed heterochromatin (null) and polycomb-associated (PcG) domains, based on the patterns of DNase hypersensitivity, H3k9me3 and H3k27me3, respectively (Additional file 1: Figure S15). This analysis reveals that active compartments typically cover both active and PcG-associated TADs, while B compartments appear more homogeneous and are composed mostly of H3k9me3-enriched heterochromatin even when considering fine-grained TAD structures rather than megabase-sized genomic blocks.

## Discussion

The recent abundance of epigenomic data for model cell types has enabled accurate modeling of the transcriptional output of human promoters, and a rigorously quantitative assessment of the most influential chromatin features underlying gene expression [16]. We have shown that it is possible to construct comparable models describing the features underlying higher-order chromatin structure, and that their predictive accuracy can be high. Our analysis exploits Hi-C datasets that have been re-analyzed, from the initial sequence read mapping onwards, identically for three different cell types. These data were collated with 35 locus-level ENCODE chromatin datasets, also processed identically, and matched across the same cell types. In common with previous studies [8,9], we observed good concordance of higher-order chromatin structure, reflected in Hi-C data, between different cell types. Random forest models summarized the important relationships among these many variables, providing insights into the quantitative contributions of locus-level chromatin features to higher-order structures. Although certain features were notably more influential in a particular cell type, the models shared overlapping constellations of informative features, allowing the cross-application of models between cell types.

Integrative analyses of locus-level chromatin data have allowed the prediction of functional chromatin states [2-5] but these states typically encompass small regions such as the enhancers examined here. The prediction of higher-order chromatin domains has received much less attention, and it was not clear until now that sufficient data existed to allow accurate predictions. Our data show that accurate predictions of Hi-C-derived eigenvector values, and the nuclear compartment domains based upon them, are entirely feasible. Strong and significant correlations are seen between cell types for a variety of human higher-order domains, delineating variation in replication timing, lamin association and nuclear compartments derived from Hi-C eigenvectors [8]. The data presented here therefore suggest that a variety of such domains could be successfully modeled. Given that the binding patterns of most human chromatin components have not yet been mapped, the models presented here are remarkably successful, though will undoubtedly improve with further data and algorithm development. These models also allowed us to probe the features underlying regions with variable higher-order structure between cell types, revealing enrichments of cell-type-specific enhancer activity, and suggesting links between functional chromatin states and higher-order domain dynamics. It is not possible to distinguish cause and effect using the current data, but it seems likely that the alterations in domain organization occur prior to enhancer activity.

The current data suggest that the contributions of certain locus-level chromatin features to higher-order structures vary between cell types. Striking examples include the strong influence of H3K9me3 in K562 leukemia cells, and EGR1 binding in H1 hESC. EGR1 is a pivotal regulator of cell fate and mitogenesis with critical roles in development and cancer [36]. The patterns of repressive H3K9me3 accumulation have been a focus in the cancer literature and have been proposed as a diagnostic marker in leukemia [37]. Similarly, the model for GM12878 (Epstein–Barr virus transformed lymphoblastoid) cells shows a disproportionate influence of ATF3 binding patterns, and ATF3 induction is a known consequence of virus-transformed cells [38]. Thus, the most cell-type-specific features in these models may be important indicators of cell-type-specific functions. These cell-type-specific features present a paradox, in view of the strong correlations in organization genome-wide across different cell types [8,9], and the demonstration that models trained in one cell type often perform well with data from other cell types. These contradictory observations are reconciled by the presence of inter-correlated clusters of features underlying A and B compartments. The shifting membership of these clusters evidently retains enough similarity between cell types to enable the cross-application of models.

Chromatin boundaries, separating TADs and nuclear compartments at different scales, also showed cell-type-specific enrichments of various locus-level chromatin features. Across cell types, the complexity of boundary composition varies considerably so that only a few features were seen consistently enriched or depleted at boundaries. Peaks associated with active promoters were notable for both TAD and compartment boundaries in all cell types. Among the most influential variables for the random forest models constructed for the two hematopoietic cell lines was the ubiquitous transcription factor YY1, which reappeared in the analysis of chromatin boundary regions. Significant enrichments of YY1 were seen at TAD and nuclear compartment boundaries in all three cell types. Thus, the same protein was implicated at the level of broad genomic binding patterns (over 1-Mb intervals) and at the level of locally enriched peaks at boundary regions (spanning 100 to 500 kb). This is intriguing as YY1 has recently been shown to co-localize with the architectural protein CTCF [39] and suggests that these proteins cooperate in the establishment of domain boundaries. The identification of such features, significantly enriched at boundary regions, provides potential targets for deletion in experimental studies further exploring the structure and function of domains (for example, [14]). Both cell-type-specific and general constituents of boundaries may have utility in the biomedical interpretation of genomic variation in noncoding regions of the genome.

## Conclusions

It has become commonplace to discuss the multi-layered, hierarchical organization of interphase chromosomes across strata ranging from nuclear compartments, down to the spectra of histone modifications and bound proteins at individual sub-genic regions. However, we lack a detailed understanding of how these strata interact. We have shown that our perspectives of features occurring at different strata can be bridged by modeling approaches, and the models produced can be used to explore the interrelationships between these different features quantitatively.

We constructed cell-type-specific models of nuclear organization, as reflected in Hi-C-derived eigenvector profiles, to discover the most influential features underlying higher-order structures. We found open and closed compartments to be well correlated with combinatorial patterns of histone modifications and DNA binding proteins, enabling accurate predictive models. These models could be cross-applied successfully between cell types highlighting constellations of common structural features associated with different nuclear compartments as expected. Dissection of the most influential variables also revealed important differences between models, consistent with the known biological contrasts among these cell types, such as the prominence of EGR1 in ESCs and H3K9me3 in the leukemia cell line. Investigation of regions showing variable nuclear organization across the three cell types under study, revealed enrichments for cell-type-specific enhancer activity, often nucleated at genes with known roles in cell-type-specific functions. Finally we used model predictions to examine boundary composition between higher-order domains across cell types. Among enrichments of a large number of factors observed at different boundaries in different cell types, CTCF and YY1 were found consistently and may cooperate to establish domain boundaries. In summary, we show that integrative modeling of large chromatin dataset collections using random forests can generate useful insights into chromosome structure and seed testable hypotheses for further experimental studies.

## Materials and methods

### Hi-C data and locus-level chromatin features

Hi-C datasets for human cell types H1 hESC [9], K562 [15] and GM12878 [40] were retrieved (Gene Expression Omnibus accession numbers: [GEO:GSE35156], [GEO:GSE18199] and [GEO:SRX030113]) and mapped to the genome (hg19/GRCh37). Iterative mapping was performed using the hiclib software package [41] and bowtie2 [42] with the very-sensitive flag. Mapped reads were then binned into contact maps and iteratively corrected [41]. The hiclib software was also used for eigenvector expansion of each intrachromosomal contact map, performed independently for each chromosome arm.

Genome-wide ChIP-seq datasets for 22 DNA binding proteins (ATF3, CEBPB, CHD1, CHD2, CMYC, CTCF, EGR1, EZH2, GABP, JUND, MAX, MXI1, NRSF, POL2, P300, RAD21, SIX5, SP1, TAF1, TBP, YY1 and ZNF143) and ten histone modifications (H3K27ac, H3K27me3, H3K36me3, H3K4me1, H3K4me2, H3K4me3, H3K79me2, H3K9ac, H3K9me3 and H4K20me1) were produced by ENCODE (July 2012 data freeze, used in [43,44]), in addition to DNase I hypersensitivity data and H2A.Z occupancy (Additional file 1: Figure S5), for each of the Tier 1 ENCODE cell lines used in this work: H1 hESC, K562 and GM12878 [4]. These data were processed using MACSv2 [45] to produce a fold-change signal relative to input chromatin and the data are available from [43]. Regional GC content was also calculated for each 1-Mb region and used in the feature modeling set (Additional file 3).

### Structural modeling and variability

Random forest regression [46] was used as implemented in the R package randomForest [47]. Parameters of *mtry*=*n*/3=12 and *ntrees*=200 were assumed as the algorithm is known to be largely insensitive [48]. Variable importance within random forest regression models was measured using the mean decrease in accuracy in the out-of-bag sample. This represents the average difference (over the forest) between the accuracy of a tree with permuted and unpermuted versions of a given variable in units of percentage mean-squared error [49]. The effectiveness of the modeling approach was measured by four different metrics. Prediction accuracy was assessed by the PCC between the predicted and observed eigenvectors (out-of-bag estimate), and the root mean-squared error of the same data. Classification error, when predictions were thresholded into *A*≥0 and *B*<0, was also calculated using accuracy (percentage correct classifications or true positives) and the area under the receiver operating characteristic (AUROC) curve. Together these give a comprehensive overview of model performance, both in terms of regression accuracy of the continuous eigenvector, and in how that same model could be used to label discrete chromatin compartments.

For cross-application of cell-type-specific models, a single random forest regression model was learned from all 1-Mb bins for a given cell type. This was then used to predict all bins from each of the other two cell types. The median absolute deviation was chosen as a robust measure of the variability in a given 1-Mb block between the three cell types. Blocks were ranked by this measure and the distribution was split into thirds that represented low variability (the third of blocks with the lowest median absolute deviation), and mid and high variability. Each subgroup was then independently modeled using the random forest approach described above. For each cell type we identified 1-Mb regions whose compartment state was altered relative to the other two. For example, if a 1-Mb bin was classified as occupying compartment A in H1 hESC and B in both K562 and GM12878, it is said to occupy an altered open compartment in H1 hESC. Chromatin state annotations were calculated from ENCODE ChromHMM/SegWay combined annotations for each cell type [5]. Annotated features were considered shared if there was an overlapping annotation in either of the two other cell types, and labeled as specific to a cell type otherwise.

### Chromatin boundaries

TAD boundaries were called using software provided by Dixon et al. [9] with recommended parameters. For the generation of locus-level feature profiles over TAD boundaries, input features were averaged into 40-kb bins spanning ±500 kb from the boundary center. For compartment boundaries, a two-state hidden Markov model was trained on the compartment eigenvector data and the Viterbi algorithm was used to infer the most likely underlying state sequence that generated the observed compartment eigenvectors. Compartment boundaries were then defined as the point of transition between different compartment types. To generate boundary profiles, locus-level features were averaged into 100-kb windows extending ±1.5 Mb either side of the boundary center.

To test for the enrichment or depletion of a chromatin feature over a given boundary, a two-tailed Mann–Whitney test was used to compare the boundary bin with the ten outermost bins of the window (five from either side). The significance level at *α*=0.01 was then Bonferroni-adjusted for multiple testing correction, and results with *P* values exceeding this threshold were deemed significantly enriched or depleted at a given boundary.

Scripts to reproduce the analyses and generate manuscripts figures are available at [50].

## Additional files

Additional file 1
**Figures S1 to S15.** Collection of supplementary figures (S1 to S15) with captions.

Additional file 2
**Tables S1 to S3.** Functional enrichments of genes located within structurally variable regions in each cell type.

Additional file 3
**cellTypeFeatureSets.** Archive containing comma-separated value (CSV) files of binned input features and compartment eigenvectors used for modeling, for each of the three cell types used in this study.

## Abbreviations

AUROC: Area under the receiver operating characteristic curve
ChIP-seq: Chromatin immunoprecipitation sequencing
ESC: Embryonic stem cell
kb: kilobases
Mb: megabases
PCC: Pearson correlation coefficient
PcG: polycomb-associated
TAD: Topological domain
