# Supplementary material for: Integrative modeling reveals the principles of multi-scale chromatin boundary formation in human nuclear organization
Source: Genome Biol. 2015 May 27;16(1):110. doi: 10.1186/s13059-015-0661-x (PMC4443654; doi:10.1186/s13059-015-0661-x)
Supplement: Additional file 1 — Figures S1 to S15. Collection of supplementary figures (S1 to S15) with captions. [file 13059_2015_661_MOESM1_ESM.pdf]

Integrative modelling reveals the principles of multi-scale boundary formation in higher order chromatin organization

Benjamin L. Moore, Stuart Aitken and Colin A. Semple

University of Edinburgh,

MRC HGU, IGMM

EH4 2XU

Additional file 1: Figures S1-S15

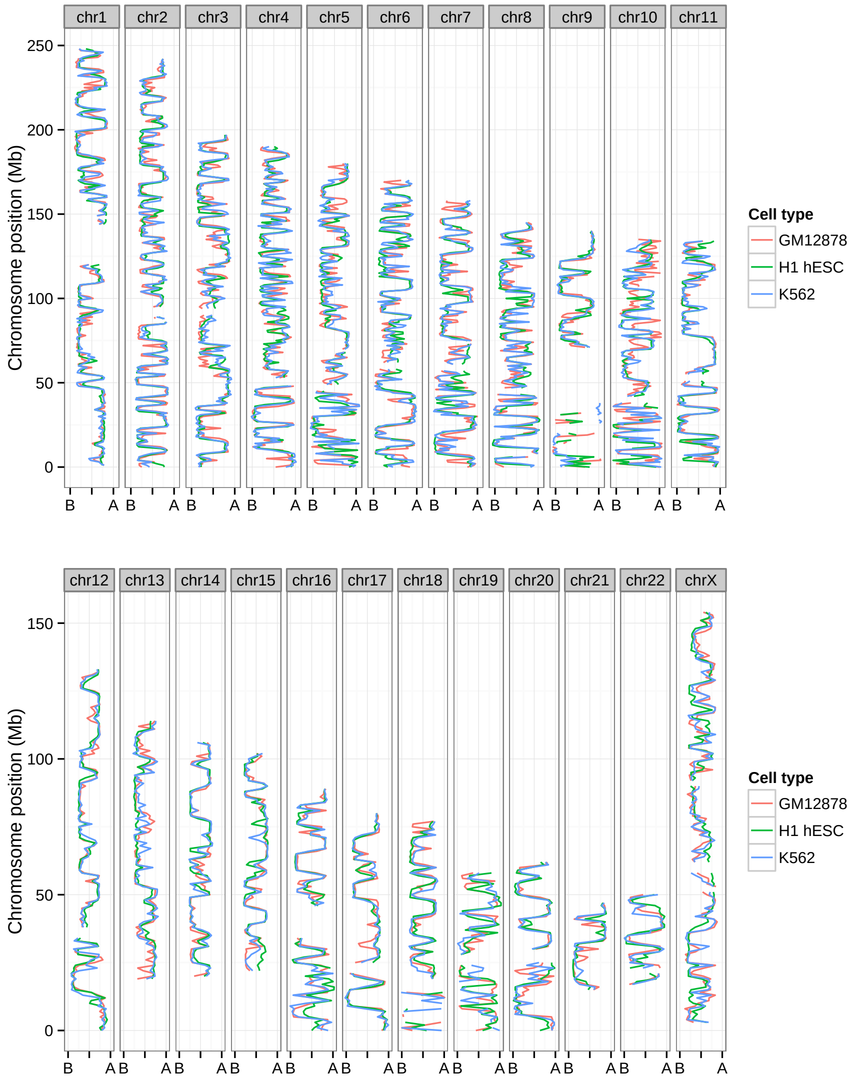

Figure S1: **Compartment eigenvectors conserved across human cell types.** In the three cell types used in this work there is a good agreement between compartment eigenvectors across the genome.

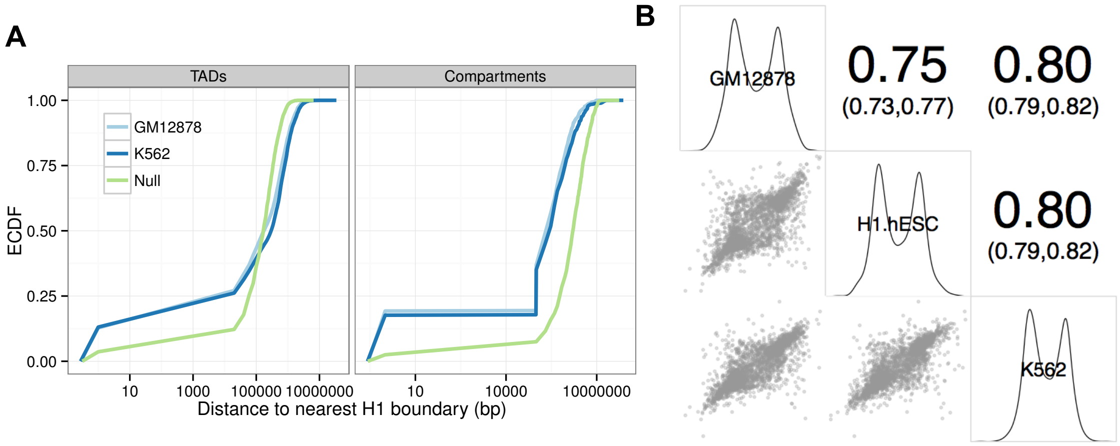

Figure S2: **TAD boundaries are significantly conserved between cell types and compartment eigenvectors are highly correlated.** (A) H1 TAD and compartment boundaries were compared with those called in GM12878 and K562 (ECDF – empirical cumulative density function). They were also compared with a null distribution calculated from randomly placed boundaries allocated at a matched resolution. (B) Pearson correlations between compartment eigenvectors in each cell type are shown along with kernel density estimates and scatterplots. Each correlation is significantly non-zero ($p \approx 0$).

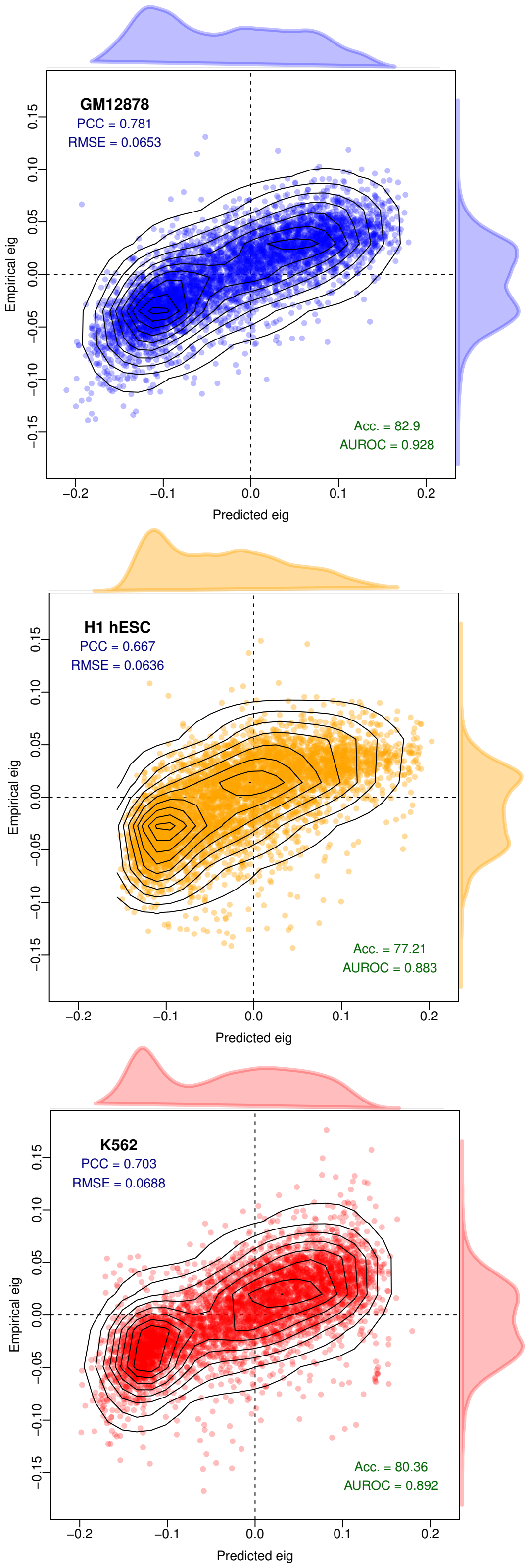

Figure S3: **Models learned at 1 Mb resolution can be applied to higher resolution datasets.** Despite having been trained on low resolution training sets, the Random Forest models generated can successfully predict compartment eigenvectors at higher resolution (100 kb, a 10× zoom). Eigenvectors at a higher resolution than this do not necessarily reflect A/B compartmentalisation.

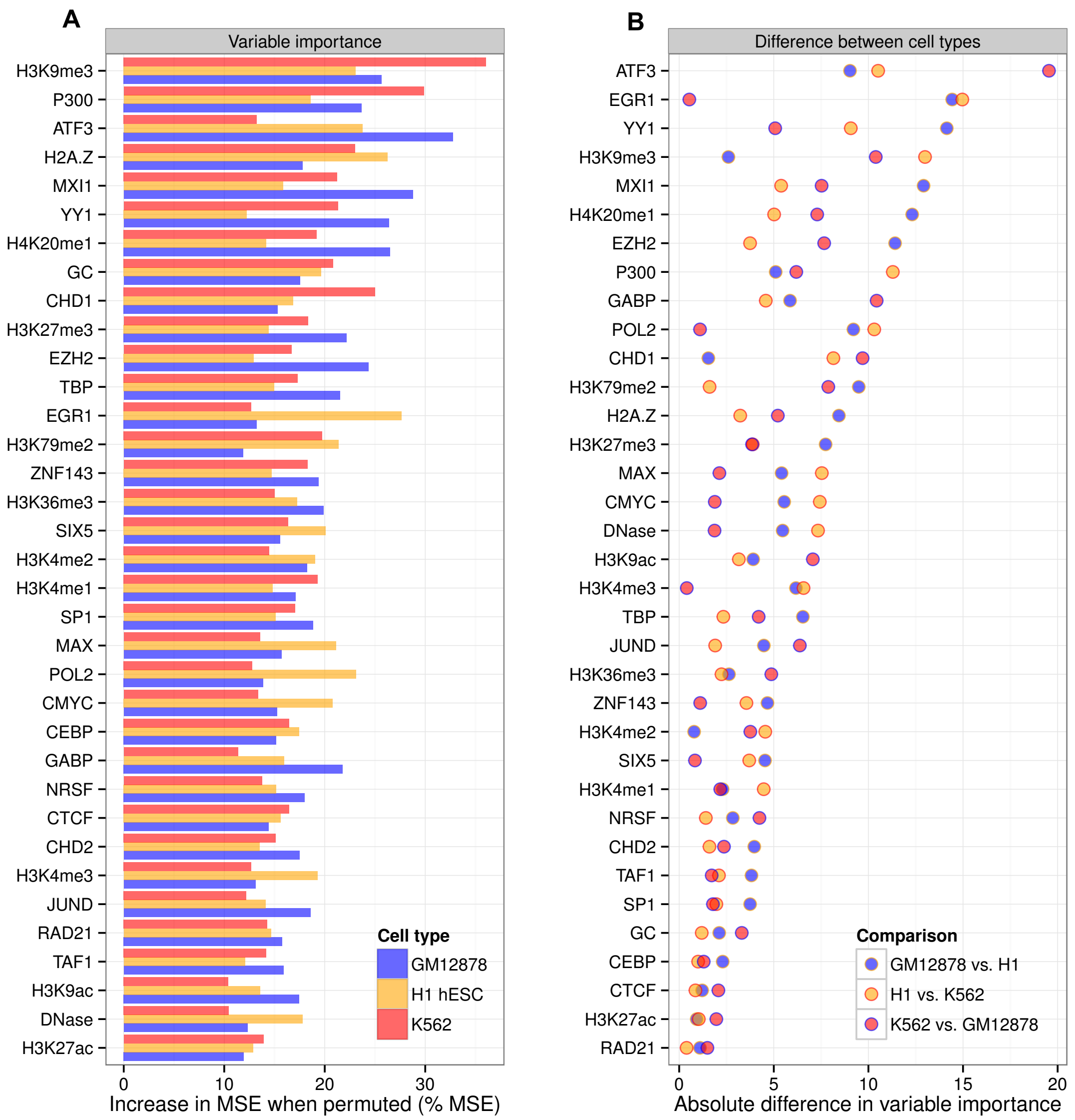

Figure S4: **Variable importance varies across features between cell types.** (A) Variable importance is calculated by permuting a given feature and measuring the decrease in accuracy (increase in MSE as a percentage of the unpermuted result) on the OOB data when building a Random Forest (Liaw and Wiener, 2002). (B) The absolute difference in this metric is then calculated between each pair of cell types used (ordered by maximum absolute difference).

Figure S5: **Correlation heatmaps of the 35 features used to model compartment eigenvectors.** The Pearson correlation coefficient (PCC) of genome-wide 1 Mb bins of each feature were pairwise correlated with each other. The features were also clustered using hierarchal clustering. The "significance" of these clusters was determined through multi-scale bootstrap resampling, with clusters that were stable across different sizes of resampling deemed significant, as implemented in the `pvclust` R package (Suzuki and Shimodaira, 2006).

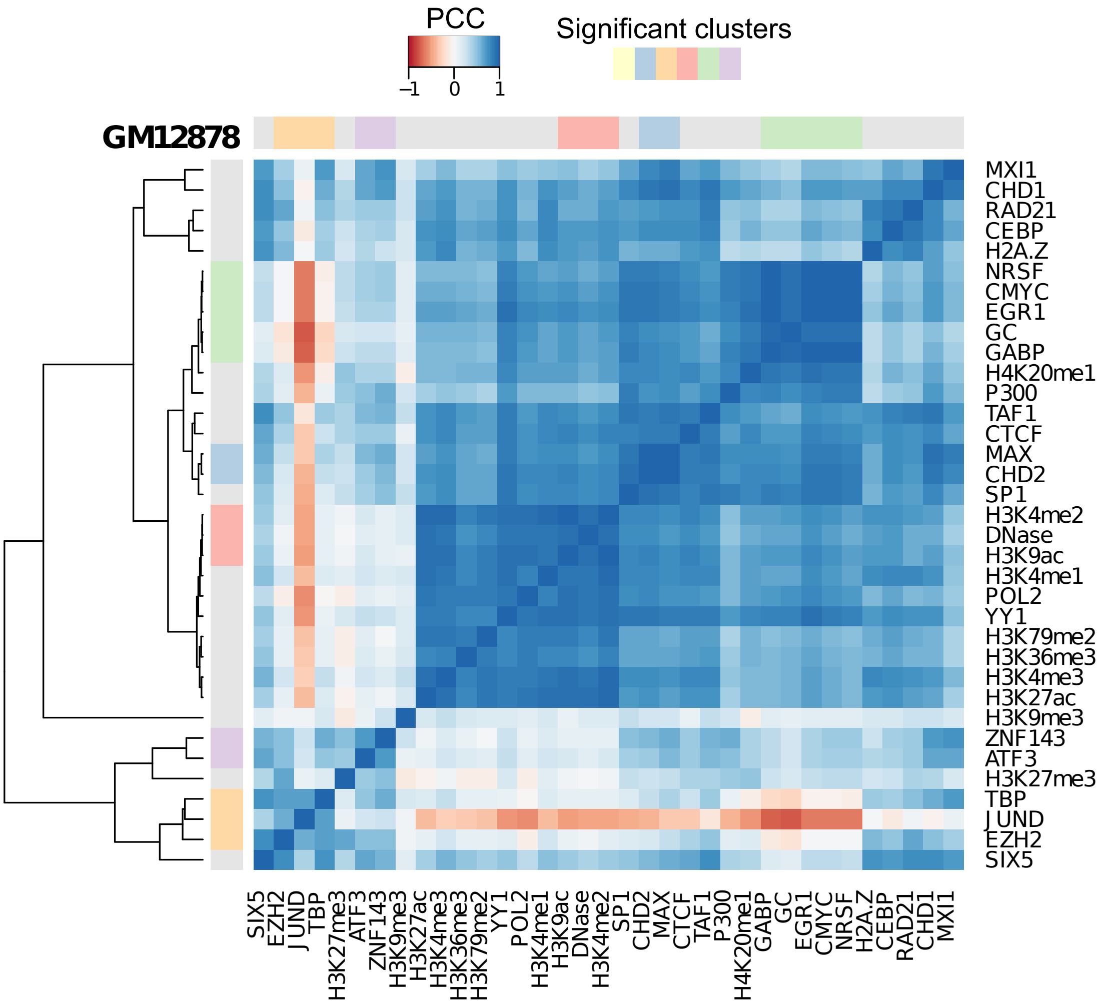

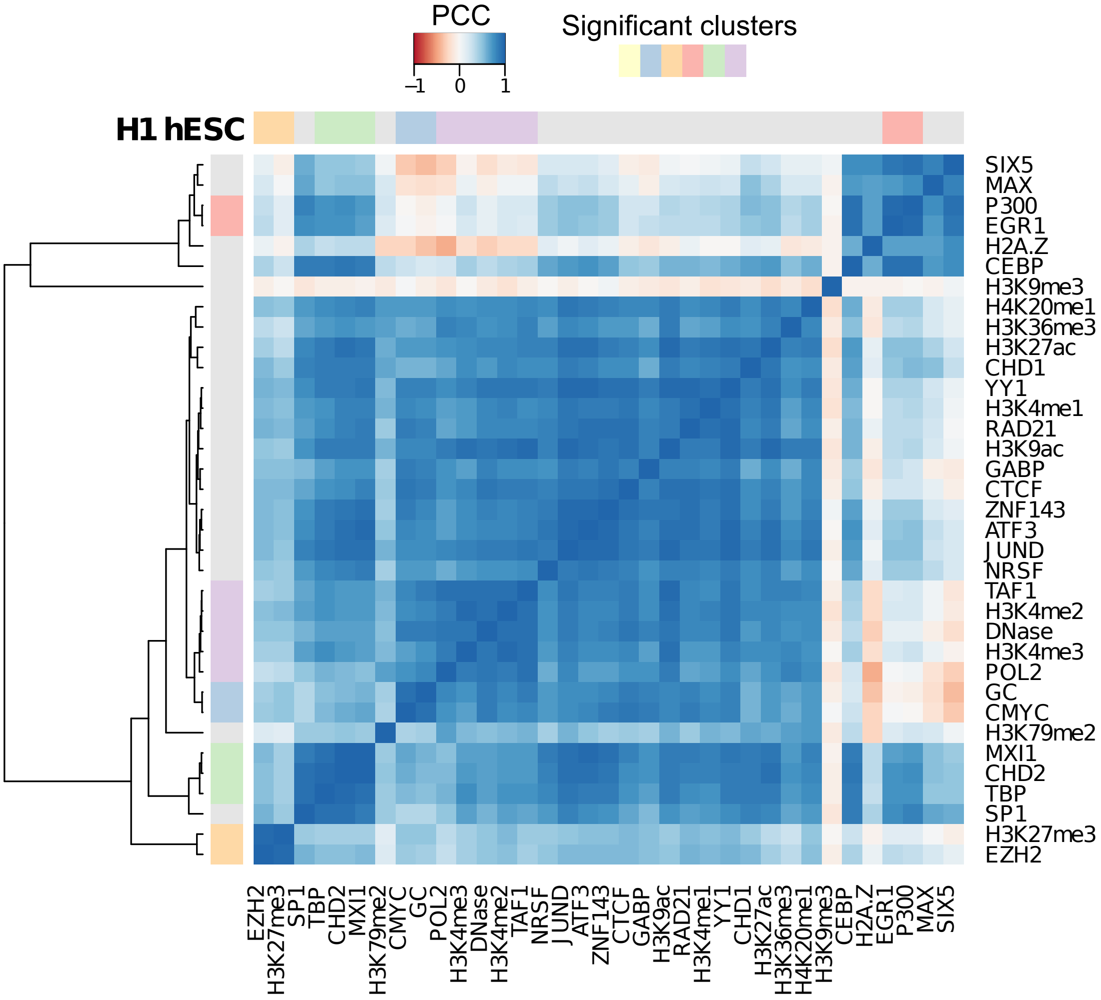

PCC
−1
0
1
Significant clusters
H1 hESC
SIX5
MAX
P300
EGR1
H2A.Z
CEBP
H3K9me3
H4K20me1
H3K36me3
H3K27ac
CHD1
YY1
H3K4me1
RAD21
H3K9ac
GABP
CTCF
ZNF143
ATF3
JUND
NRSF
TAF1
H3K4me2
DNase
H3K4me3
POL2
GC
CMYC
H3K79me2
MXI1
CHD2
TBP
SP1
H3K27me3
EZH2

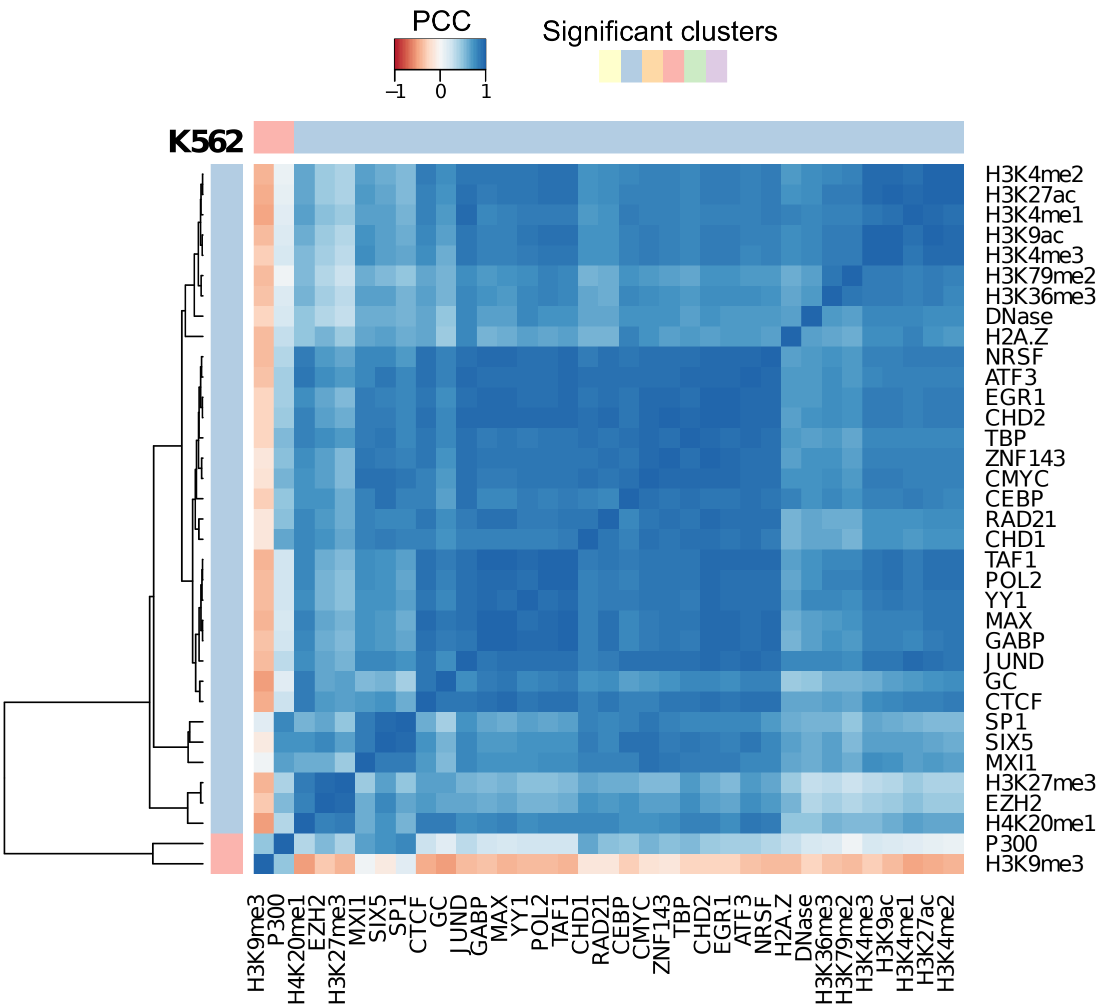
PCC
−1
0
1
Significant clusters
K562
H3K4me2
H3K27ac
H3K4me1
H3K9ac
H3K4me3
H3K79me2
H3K36me3
DNase
H2A.Z
NRSF
ATF3
EGR1
CHD2
TBP
ZNF143
CMYC
CEBP
RAD21
CHD1
TAF1
POL2
YY1
MAX
GABP
JUND
GC
CTCF
SP1
SIX5
MXI1
H3K27me3
EZH2
H4K20me1
P300
H3K9me3

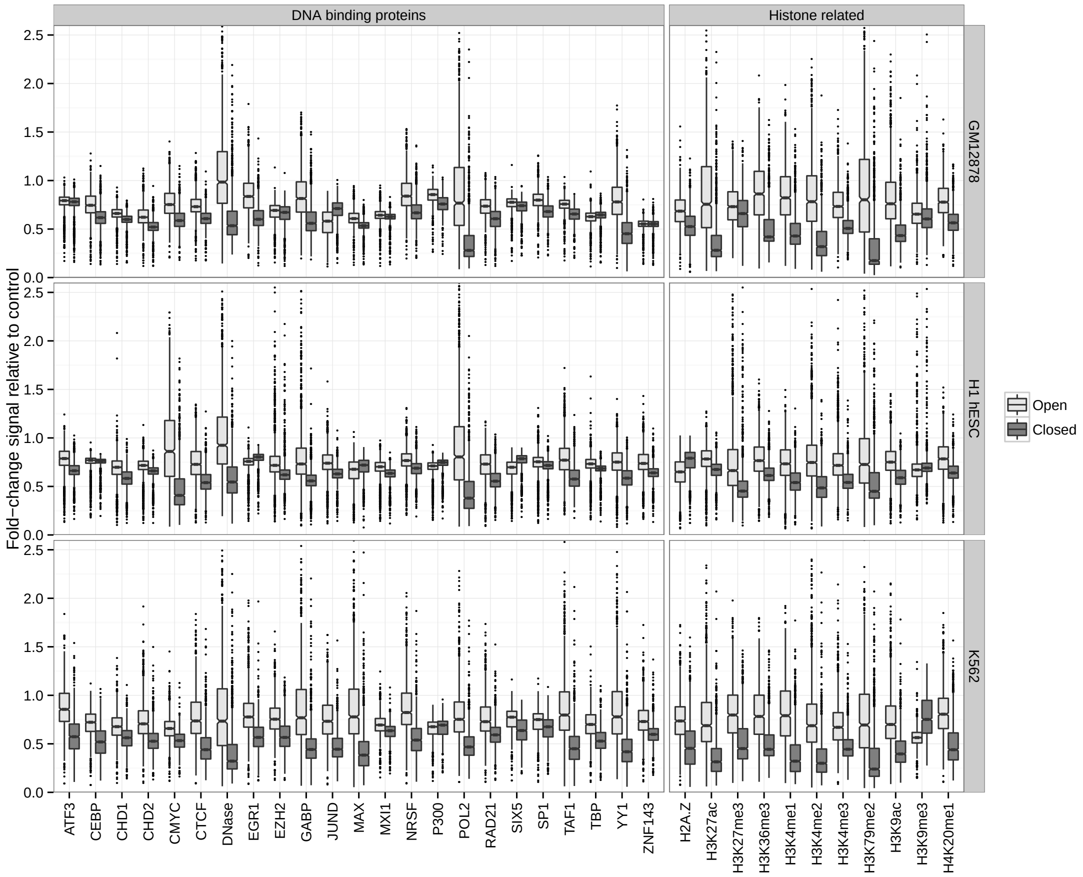

Figure S6: **Distribution of input features in active and inactive compartments.** Notched boxplots summarise the distribution of each feature over 1 Mb bins in open (A) and closed (B) compartments genome-wide.

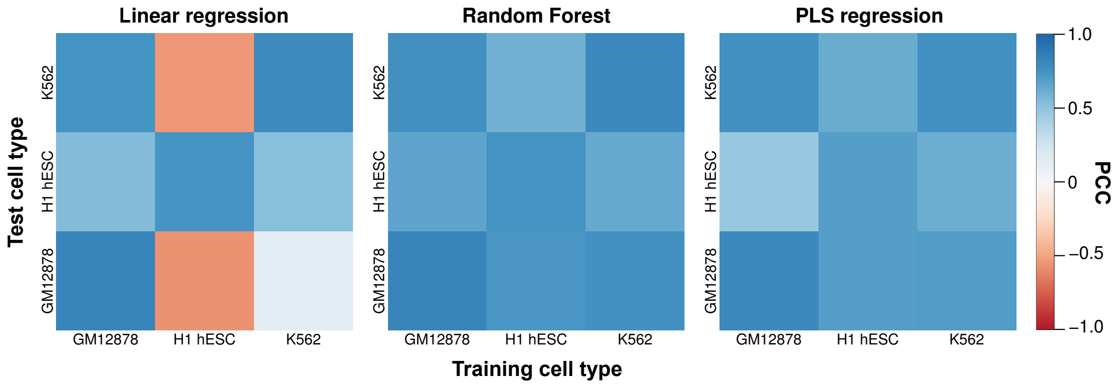

Figure S7: **Comparison of Random Forest performance with other modelling approaches.** Heatmaps show the Pearson correlation coefficient between predicted and observed compartment eigenvectors genome-wide for three regression techniques: multiple linear regression (LM), Random Forest (RF) and partial least squares (PLS). Cell type specific predictions were assessed using cross-validation (linear regression and PLS) and OOB estimates (Random Forest), while cross-applied models were learnt from the full dataset of the training cell type. For PLS, the optimal number of components was also selected using cross-validation. Cell type specific models (heatmap diagonal) can be learnt with similar success via each method (mean PCC for cell type specific models: LM 0.787, RF 0.790, PLS 0.750) but on cross-application RF and PLS prove much more generalisable (mean PCC over cross-applications: LM 0.139, RF 0.689, PLS 0.641).

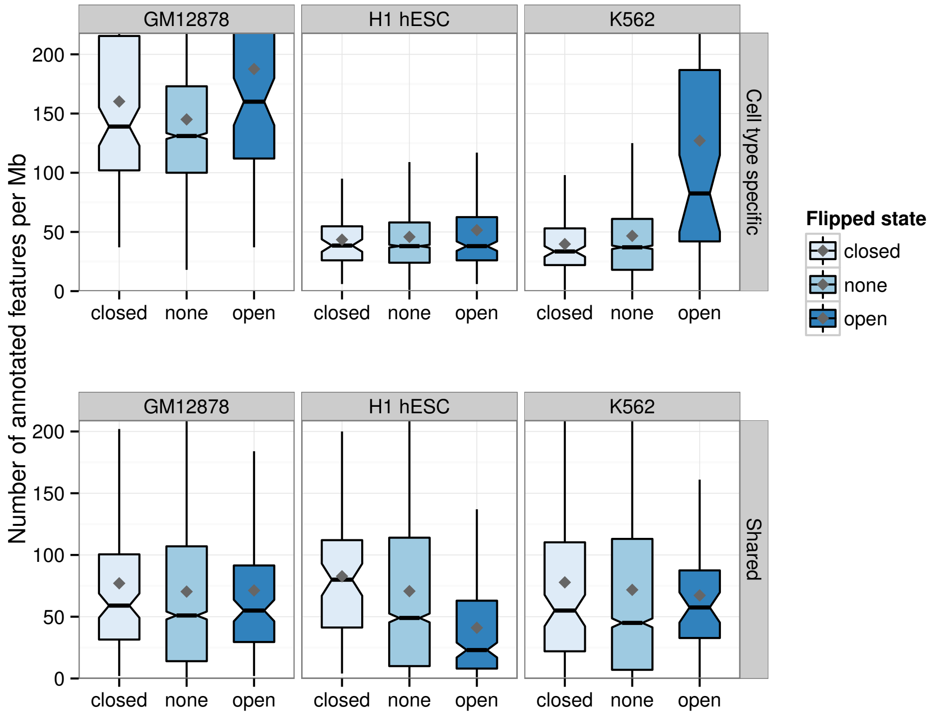

Figure S8: **Enrichment of cell type specific transcribed regions in areas of variable higher order chromatin organisation.** Following Figure 4b, the number of predicted transcribed regions per cell type are shown for the blocks which are: “open”: A in the given cell type but B in the other two, “closed” *vice versa* with B compartments, and “none” blocks which are in the same compartment state across cell types. Distributions of all chromatin states are shown in Figure S9.

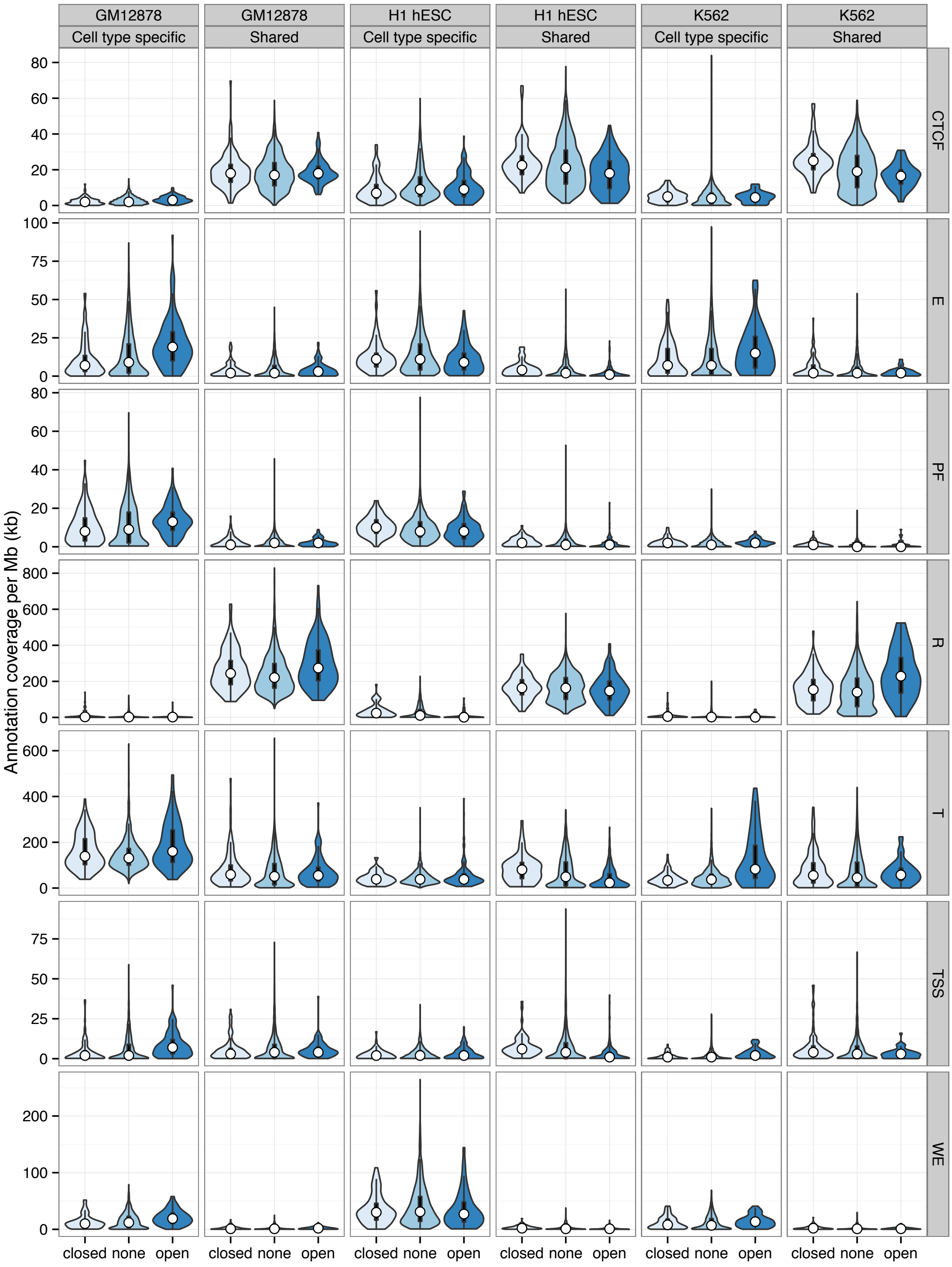

Figure S9: **Distributions of features across all predicted chromatin states in regions of variable higher order structure.** Following Figure 4b and S8, the distributions of the summed coverage of predicted chromatin states in each Mb per cell type are shown as bean plots. Predicted chromatin states are those from Hoffman et al. (2013) and are labelled as: TSS: promoter and TSS; PF: promoter flanking region; E: enhancer; WE: weak enhancer or *cis*-regulatory element; CTCF: CTCF enriched element; T: transcribed region; R: repressed or low-activity.

Figure S10: **Regions of variable higher order structure in each cell type.** Here we show the top five regions (by number of predicted enhancers) of variable compartment structure between the three cell types under study, i.e. Mb blocks which in one cell type are in the active "A" compartment, while classified in the "B" inactive compartment state in the other two cell types. Panels are labelled: (A) GM12878 open compartments (B) H1 hESC (C) K562. For complete legends, refer to Figure 5. Genome browser screenshots were produced using UCSC Genome Browser (Hinrichs et al., 2006) with the hg19 reference genome, and show (in order): UCSC genes, then for each cell type: compartment eigenvectors (recalculated in this work); ChromHMM / SegWay combined predicted chromatin states (ENCODE, 2012) and FAIRE peaks. Below this is a track of H3K27ac signal, colour-coded for each cell type: red (GM12878), blue (K562) and orange (H1 hESC).

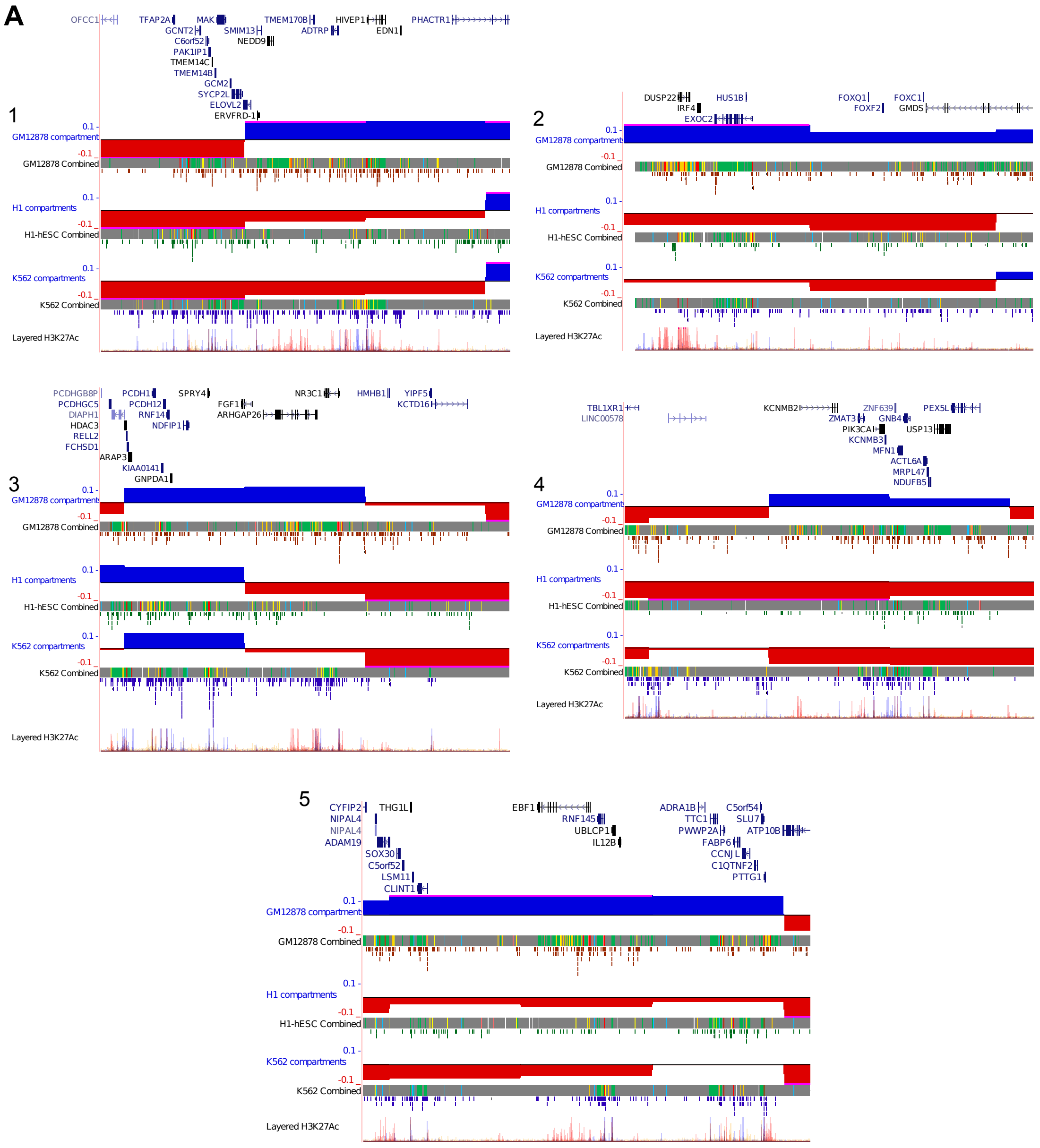

B

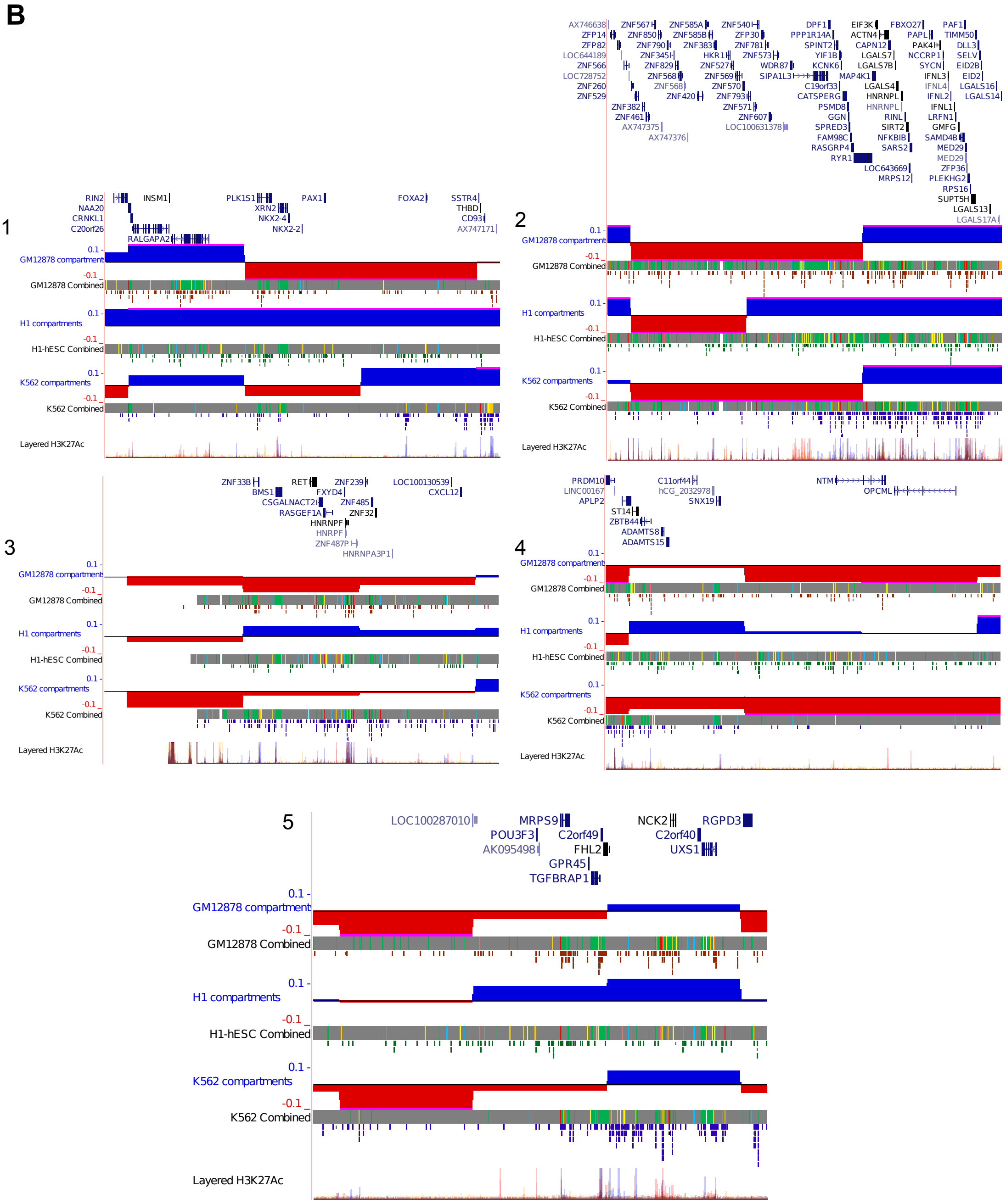
1
2
3
4
5
GM12878 compartment
GM12878 Combined
H1 compartments
H1-hESC Combined
K562 compartments
K562 Combined
Layered H3K27Ac
LOC100287010
MRPS9
NCK2
RGPD3
POU3F3
C2orf49
C2orf40
AK095498
FHL2
UXS1
GPR45
TGFBRAP1

C

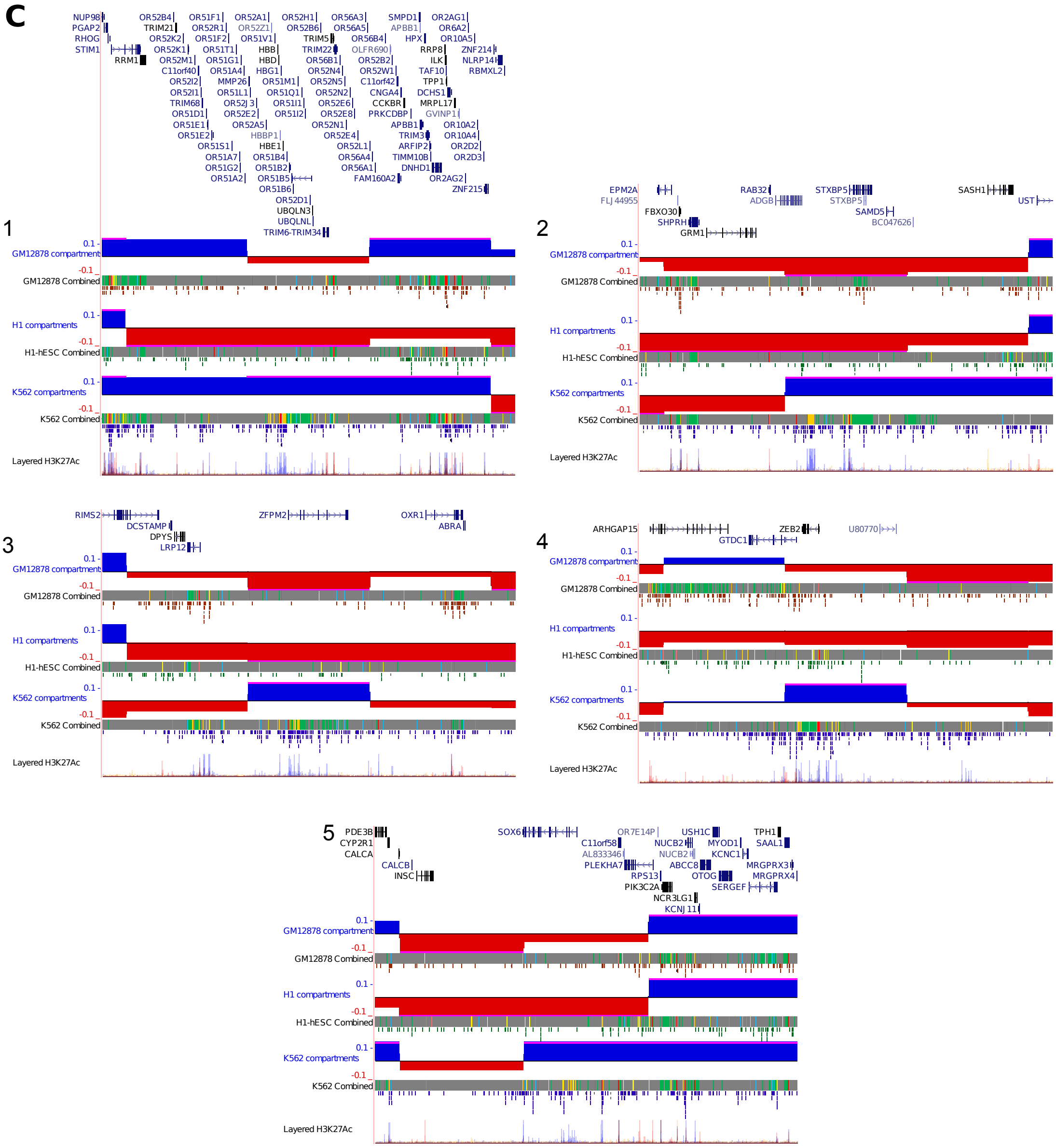

1
NUP98 PGAP2 RHOG STIM1 RRM1
OR52B4 TRIM21 OR52K2 OR52K1 OR52M1 C11orf40 OR52I2 OR52I1 TRIM68 OR51D1 OR51E1 OR51E2
OR51F1 OR52R1 OR51F2 OR51T1 OR51G1 OR51A4 MMP26 OR51L1 OR52J3 OR52E2 OR52A5 HBBP1 OR51S1 OR51A7 OR51G2 OR51A2
OR52A1 OR52Z1 OR51V1 HBB HBD HBG1 OR51M1 OR51Q1 OR51I1 OR51I2 HBE1 OR51B4 OR51B2 OR51B5 OR51B6 OR52D1 UBQLN3 UBQLNL TRIM6-TRIM34
OR52H1 OR52B6 TRIM5 TRIM22 OR56B1 OR52N4 OR52N5 OR52N2 OR52E6 OR52E8 OR52N1 OR52E4 OR52L1 OR56A4 OR56A1
OR56A3 OR56A5 OR56B4 OLFR690 OR52B2 OR52W1 C11orf42 CNGA4 CCKBR PRKCDBP APBB1 TRIM3 ARFIP2 TIMM10B DNHD1 FAM160A2
SMPD1 APBB1 HPX RRP8 ILK TAF10 TPP1 DCHS1 MRPL17 GVINP1
OR2AG1 OR6A2 OR10A5 ZNF214 NLRP14 RBMXL2 OR10A2 OR10A4 OR2D2 OR2D3 OR2AG2 ZNF215
2
EPM2A FLJ 44955 FBXO30 SHPRH GRM1 RAB32 ADGB STXBP5 STXBP5 SAMD5 BC047626 SASH1 UST
3
RIMS2 DCSTAMP DPYS LRP12 ZFPM2 OXR1 ABRA
4
ARHGAP15 GTDC1 ZEB2 U80770
5
PDE3B CYP2R1 CALCA CALCB INSC SOX6 C11orf58 AL833346 PLEKHA7 RPS13 PIK3C2A NCR3LG1 KCNJ11 OR7E14P NUCB2 NUCB2 ABCC8 USH1C OTOG MYOD1 KCNC1 SERGEF TPH1 SAAL1 MRGPRX3 MRGPRX4
0.1
-0.1
GM12878 compartment
GM12878 Combined
H1 compartments
H1-hESC Combined
K562 compartments
K562 Combined
Layered H3K27Ac

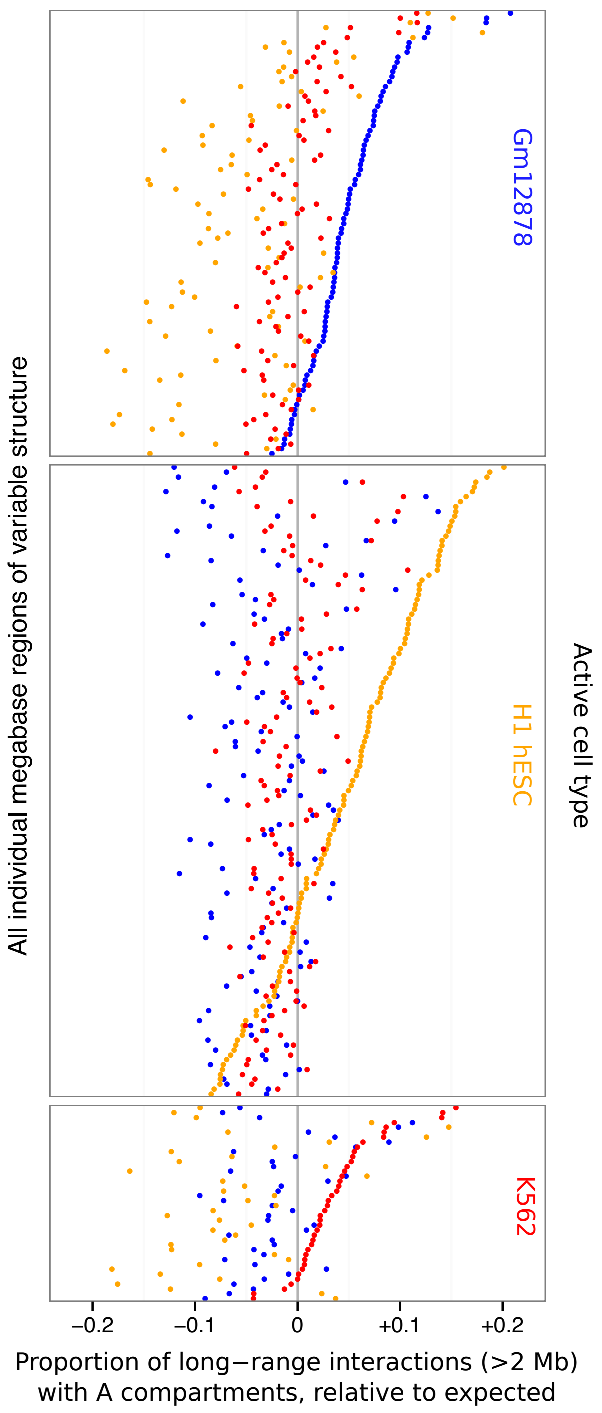

Figure S11: **Regions of variable higher order structure change their genome-wide contact profiles to favour active compartments.** Genome-wide normalised contacts were summed for each region of variable structure and the relative proportion of those that were with active / A compartments is shown across the three cell types used in this study. Proportions were subtracted from the genome-wide average per cell type, such that positive values indicate a greater than expected interaction bias with active compartments. In the majority of individual megabase regions there's an observable A compartment contact bias in the cell type in which it is active, relative to the two other cell types in which the region is in an inactive state. These results are also summarised in a boxplot (Figure 5B).

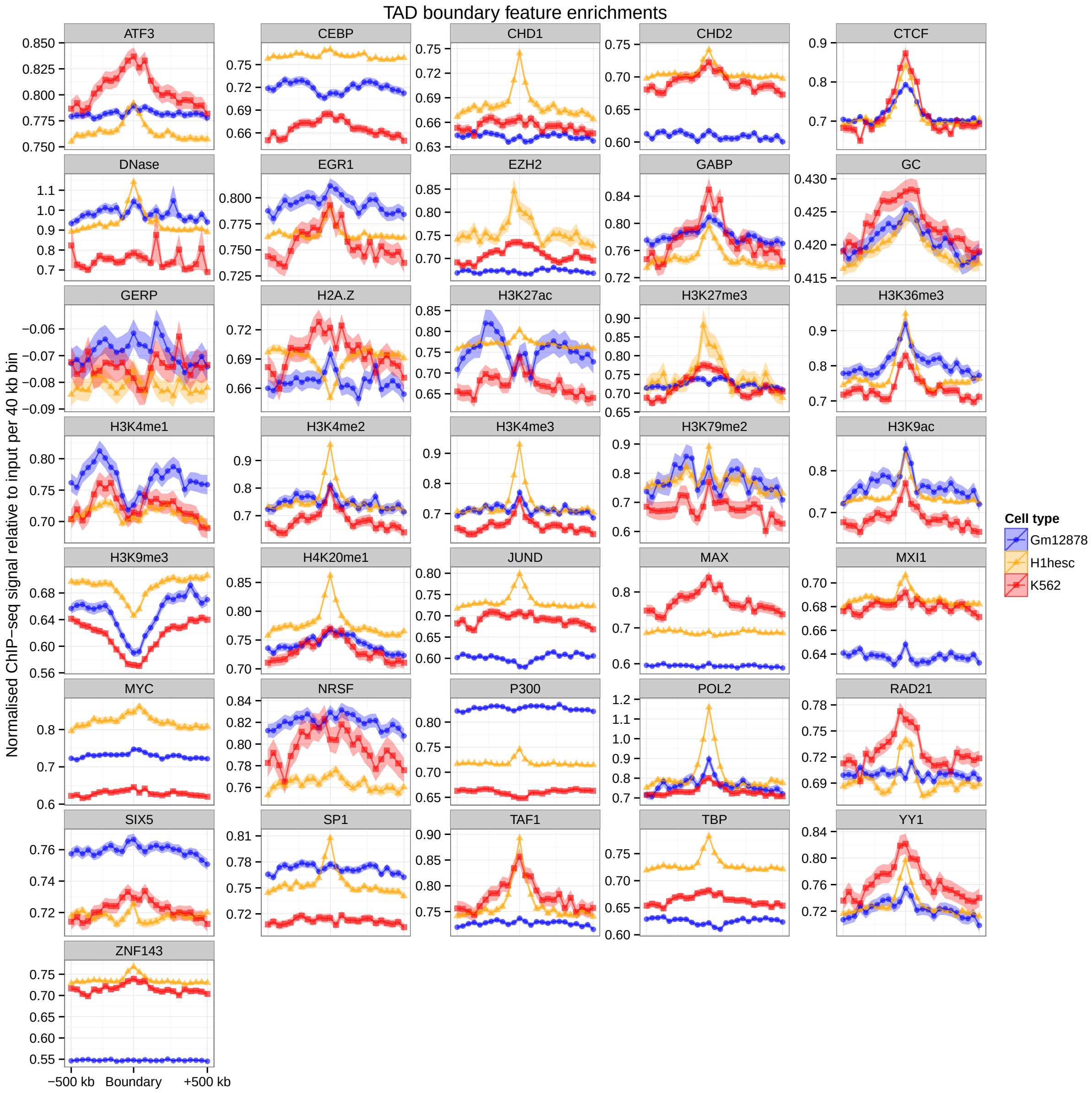

Figure S12: **All TAD boundary profiles** Average feature profiles are shown for 36 data series (as in Figure 6a). The average value of all boundaries is shown per 40 kb bin, ±500 kb around the boundary. Ribbons show ±1 standard error around the mean.

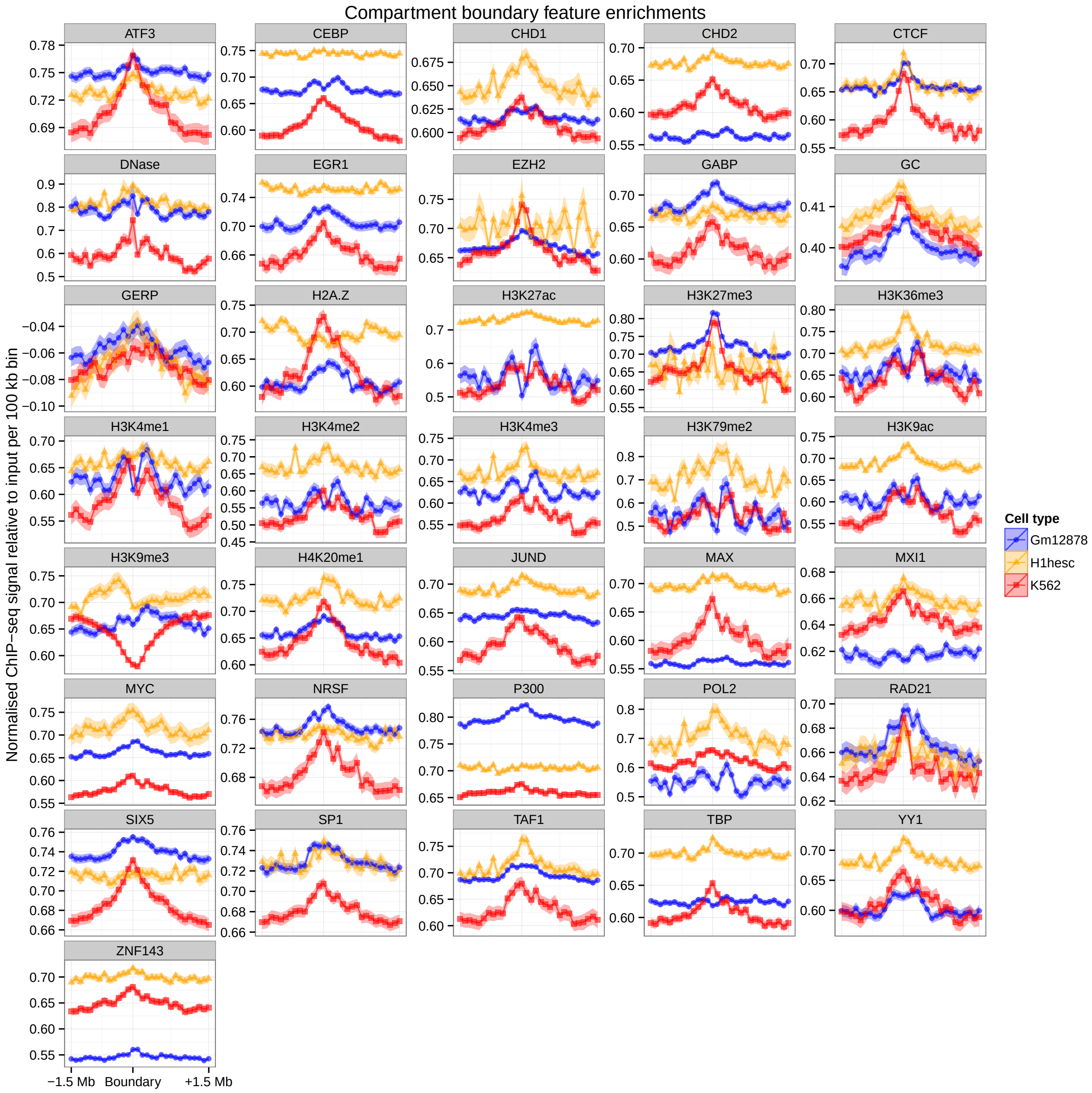

Figure S13: **All compartment boundary profiles** Average feature profiles are shown for 36 data series (as in Figure 6a). The average value of all boundaries is shown per 100 kb bin, ±1.5 Mb around the boundary. Ribbons show ±1 standard error around the mean.

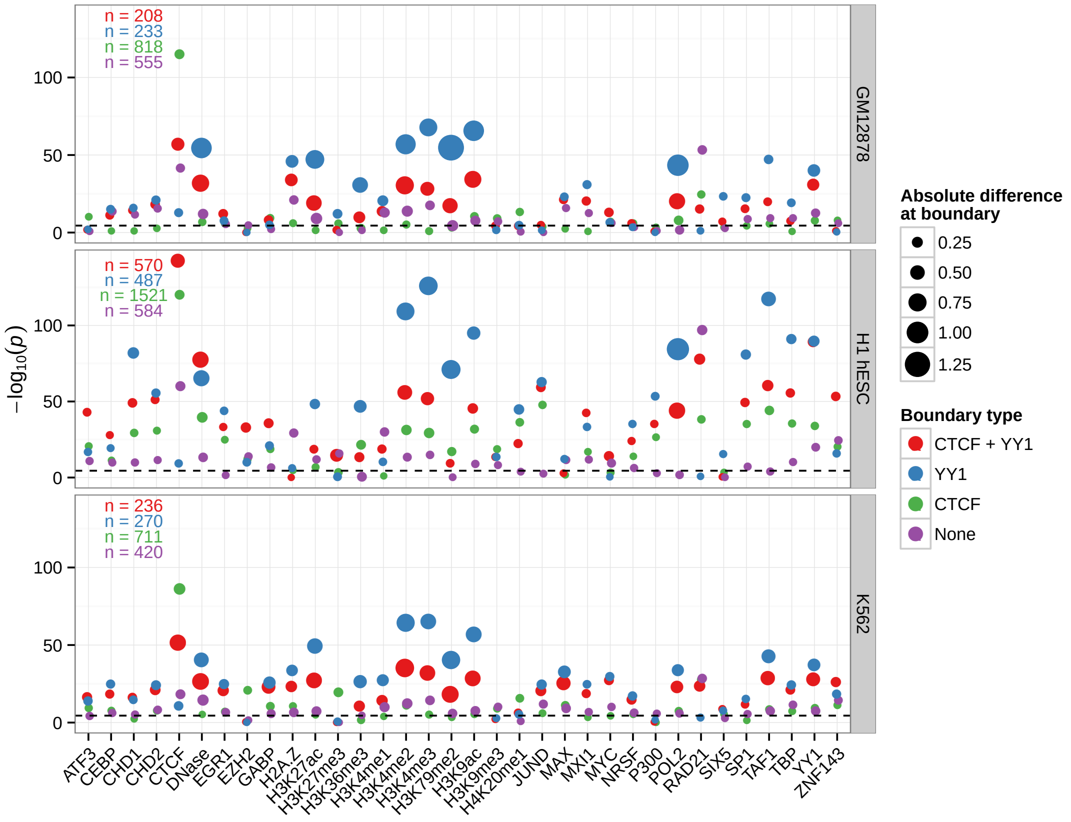

Figure S14: **TAD boundary enrichments differ for those marked with CTCF, YY1 or both.** TAD boundary feature enrichments are shown (as in Figure 6b) for TAD boundaries which have been split into classes based on specific enrichments: CTCF and YY1 groups are those boundary bins (40 kb) featuring at least one ENCODE region peak (ENCODE, 2012) for their respective features, while CTCF + YY1 is the group of boundaries which had one or more overlapping peaks for these two factors. The "none" group encompasses boundaries with neither a CTCF or YY1 region peak called, note that these boundaries lacking called peaks can still be enriched for their respective features in terms of mean ChIP-seq signal. The significance of enrichment or depletion ($-\log_{10} p$ two-tailed Mann-Whitney test) of a feature was calculated as the boundary bin relative to the 10 most peripheral bins (5 either side). Points are scaled by the absolute mean difference in signal over the boundary relative to the mean of peripheral bins.

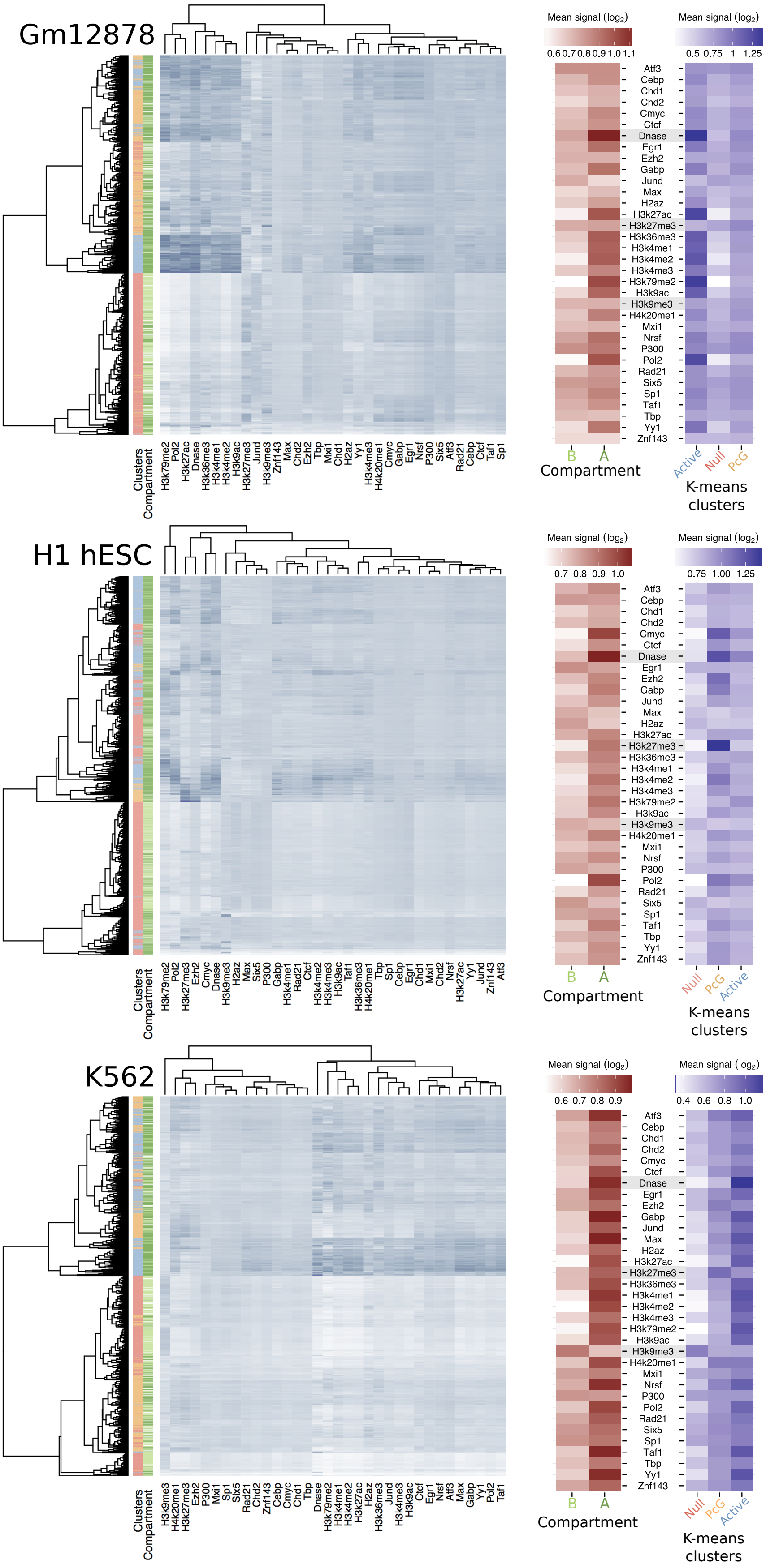

Figure S15: **TADs reflect epigenetic domains.** Following the *Drosophila* results of Sexton et al. (2012), clustering of TAD domains by mean $\log_2$ signal of 34 ENCODE features distinguishes “null”, “active” and polycomb-associated (PcG) domains, as well as reflecting the encompassing A/B compartments.

References

ENCODE. 2012. An integrated encyclopedia of DNA elements in the human genome. *Nature* **489**: 57–74.

Hinrichs AS, Karolchik D, Baertsch R, Barber GP, Bejerano G, Clawson H, Diekhans M, Furey TS, Harte RA, Hsu F, et al.. 2006. The UCSC Genome Browser Database: update 2006. *Nucleic acids research* **34**: D590–8.

Hoffman MM, Ernst J, Wilder SP, Kundaje A, Harris RS, Libbrecht M, Giardine B, Ellenbogen PM, Bilmes Ja, Birney E, et al.. 2013. Integrative annotation of chromatin elements from ENCODE data. *Nucleic acids research* **41**: 827–41.

Liaw A and Wiener M. 2002. Classification and Regression by randomForest. *R News* **2**: 18–22.

Sexton T, Yaffe E, Kenigsberg E, Bantignies F, Leblanc B, Hoichman M, Parrinello H, Tanay A, and Cavalli G. 2012. Three-dimensional folding and functional organization principles of the Drosophila genome. *Cell* **148**: 458–72.

Suzuki R and Shimodaira H. 2006. Pvclust: an R package for assessing the uncertainty in hierarchical clustering. *Bioinformatics (Oxford, England)* **22**: 1540–2.

Code to replicate all analysis and figures is available at: `https://github.com/blmoore/3dgenome`
