# Supplementary material for: Integrative modeling reveals the principles of multi-scale chromatin boundary formation in human nuclear organization
Source: Genome Biol. 2015 May 27;16(1):110. doi: 10.1186/s13059-015-0661-x (PMC4443654; doi:10.1186/s13059-015-0661-x)
Supplement: Additional file 2 — Tables S1 to S3. Functional enrichments of genes located within structurally variable regions in each cell type. [file 13059_2015_661_MOESM2_ESM.pdf]

Additional file 2: Tables S1-S3 Functional enrichments of genes found in structurally variable regions per cell type, filtered by FDR < .05. Enrichments were calculated using DAVID (Huang and Lempicki, 2008; Huang et al., 2009).

Table 1: Gm12878 functional enrichments in variable regions.

Category	Term	Count	%	Fold Enrichment	p-value	FDR
GOTERM_CC_FAT	GO:0005882 intermediate filament	36	4.20	4.90	6.42E-15	8.95E-12
GOTERM_CC_FAT	GO:0045111 intermediate filament cytoskeleton	36	4.20	4.79	1.35E-14	1.87E-11
SP_PIR_KEYWORDS	keratin	31	3.62	5.64	1.72E-14	2.47E-11
INTERPRO	IPR007951:PMG	11	1.28	25.11	9.80E-14	1.56E-10

Table 2: H1 hESC functional enrichments in variable regions.

Category	Term	Count	%	Fold Enrichment	*p*-value	FDR
PIR_SUPERFAMILY	PIRSF003152:G protein-coupled olfactory receptor, class II	116	10.55	6.64	3.25E-68	4.41E-65
INTERPRO	IPR000725:Olfactory receptor	116	10.55	6.53	7.58E-63	1.21E-59
SP_PIR_KEYWORDS	olfaction	116	10.55	6.40	2.07E-61	2.97E-58
GOTERM_MF_FAT	GO:0004984 olfactory receptor activity	116	10.55	6.13	1.30E-60	1.97E-57
GOTERM_BP_FAT	GO:0007608 sensory perception of smell	117	10.64	5.96	1.91E-59	3.35E-56
GOTERM_BP_FAT	GO:0007606 sensory perception of chemical stimulus	118	10.73	5.37	1.71E-54	3.01E-51
KEGG_PATHWAY	hsa04740:Olfactory transduction	108	9.82	4.94	8.72E-51	1.03E-47
SP_PIR_KEYWORDS	sensory transduction	125	11.36	4.58	2.61E-48	3.74E-45
INTERPRO	IPR017452:GPCR, rhodopsin-like superfamily	131	11.91	4.03	1.40E-44	2.24E-41
INTERPRO	IPR000276:7TM GPCR, rhodopsin-like	131	11.91	4.02	1.68E-44	2.68E-41
PIR_SUPERFAMILY	PIRSF800006:rhodopsin-like G protein-coupled receptors	131	11.91	3.63	5.04E-43	6.85E-40
GOTERM_BP_FAT	GO:0007600 sensory perception	138	12.55	3.54	4.78E-41	8.40E-38
SP_PIR_KEYWORDS	g-protein coupled receptor	136	12.36	3.62	1.69E-40	2.42E-37
GOTERM_BP_FAT	GO:0050890 cognition	143	13.00	3.23	5.34E-38	9.38E-35
SP_PIR_KEYWORDS	transducer	137	12.45	3.39	1.48E-37	2.12E-34
GOTERM_BP_FAT	GO:0050877 neurological system process	163	14.82	2.72	3.85E-34	6.76E-31
GOTERM_BP_FAT	GO:0007186 G-protein coupled receptor protein signaling pathway	148	13.45	2.77	1.36E-31	2.40E-28
SP_PIR_KEYWORDS	receptor	172	15.64	2.31	3.72E-26	5.33E-23
GOTERM_BP_FAT	GO:0007166 cell surface receptor linked signal transduction	188	17.09	2.06	8.02E-24	1.41E-20
SP_PIR_KEYWORDS	cell membrane	198	18.00	1.86	5.96E-19	8.52E-16
UP_SEQ_FEATURE	topological domain:Extracellular	227	20.64	1.72	1.26E-17	2.20E-14
UP_SEQ_FEATURE	topological domain:Cytoplasmic	250	22.73	1.52	1.13E-12	1.98E-09
UP_SEQ_FEATURE	disulfide bond	211	19.18	1.56	9.11E-12	1.60E-08
SP_PIR_KEYWORDS	disulfide bond	214	19.45	1.52	6.20E-11	8.88E-08
UP_SEQ_FEATURE	glycosylation site:N-linked (GlcNAc...)	285	25.91	1.41	7.31E-11	1.28E-07
GOTERM_CC_FAT	GO:0005886 plasma membrane	255	23.18	1.37	1.26E-09	1.77E-06
SP_PIR_KEYWORDS	glycoprotein	289	26.27	1.37	1.83E-09	2.61E-06
GOTERM_CC_FAT	GO:0016021 integral to membrane	328	29.82	1.27	9.34E-09	1.31E-05
SP_PIR_KEYWORDS	transmembrane	317	28.82	1.31	1.37E-08	1.96E-05
UP_SEQ_FEATURE	transmembrane region	314	28.55	1.31	2.03E-08	3.56E-05
GOTERM_CC_FAT	GO:0031224 intrinsic to membrane	333	30.27	1.24	7.49E-08	1.05E-04
SMART	SM00355:ZnF_C2H2	69	6.27	1.86	4.23E-07	5.43E-04
UP_SEQ_FEATURE	zinc finger region:C2H2-type 5	55	5.00	2.08	5.12E-07	8.99E-04
UP_SEQ_FEATURE	zinc finger region:C2H2-type 4	57	5.18	2.01	8.49E-07	0.0015
INTERPRO	IPR013087:Zinc finger, C2H2-type/integrase, DNA-binding	59	5.36	1.94	1.73E-06	0.0028
UP_SEQ_FEATURE	zinc finger region:C2H2-type 2	58	5.27	1.95	1.73E-06	0.0030
SP_PIR_KEYWORDS	membrane	372	33.82	1.21	2.69E-06	0.0038
INTERPRO	IPR015880:Zinc finger, C2H2-like	69	6.27	1.78	4.09E-06	0.0065
UP_SEQ_FEATURE	zinc finger region:C2H2-type 8	44	4.00	2.14	4.13E-06	0.0073
UP_SEQ_FEATURE	zinc finger region:C2H2-type 3	58	5.27	1.90	4.43E-06	0.0078
UP_SEQ_FEATURE	zinc finger region:C2H2-type 7	46	4.18	2.06	5.87E-06	0.0103
INTERPRO	IPR007087:Zinc finger, C2H2-type	67	6.09	1.75	9.19E-06	0.0147
UP_SEQ_FEATURE	zinc finger region:C2H2-type 6	48	4.36	1.99	9.83E-06	0.0173

Table 3: K562 functional enrichments in variable regions.

Category	Term	Count	%	Fold Enrichment	p-value	FDR
PIR_SUPERFAMILY	PIRSF038651:G protein-coupled olfactory receptor, class I	26	7.08	24.94	7.86E-30	8.99E-27
GOTERM_MF_FAT	GO:0004984 olfactory receptor activity	40	10.90	6.12	7.39E-20	1.01E-16
INTERPRO	IPR000725:Olfactory receptor	39	10.63	6.18	3.00E-19	4.29E-16
SP_PIR_KEYWORDS	olfaction	39	10.63	6.15	4.55E-19	6.09E-16
GOTERM_BP_FAT	GO:0007608 sensory perception of smell	39	10.63	5.48	1.19E-17	1.94E-14
SP_PIR_KEYWORDS	sensory transduction	44	11.99	4.60	8.72E-17	1.44E-13
GOTERM_BP_FAT	GO:0007606 sensory perception of chemical stimulus	39	10.63	4.89	6.32E-16	1.09E-12
KEGG_PATHWAY	hsa04740:Olfactory transduction	38	10.35	4.58	6.87E-16	7.22E-13
INTERPRO	IPR017452:GPCR, rhodopsin-like superfamily	43	11.72	3.72	2.96E-13	4.23E-10
INTERPRO	IPR000276:7TM GPCR, rhodopsin-like	43	11.72	3.72	3.10E-13	4.43E-10
SP_PIR_KEYWORDS	transducer	46	12.53	3.26	4.97E-12	6.65E-09
SP_PIR_KEYWORDS	g-protein coupled receptor	44	11.99	3.35	6.34E-12	8.48E-09
PIR_SUPERFAMILY	PIRSF800006:rhodopsin-like G protein-coupled receptors	42	11.44	3.26	6.34E-12	7.26E-09
GOTERM_BP_FAT	GO:0007600 sensory perception	45	12.26	3.18	1.10E-11	1.80E-08
GOTERM_BP_FAT	GO:0050890 cognition	46	12.53	2.87	1.87E-10	3.07E-07
UP_SEQ_FEATURE	zinc finger region:C2H2-type 10	27	7.36	4.64	1.94E-10	3.10E-07
UP_SEQ_FEATURE	zinc finger region:C2H2-type 1; degenerate	17	4.63	8.23	2.35E-10	3.77E-07
GOTERM_BP_FAT	GO:0007186 G-protein coupled receptor protein signaling pathway	51	13.90	2.63	2.87E-10	4.70E-07
UP_SEQ_FEATURE	zinc finger region:C2H2-type 11	25	6.81	4.91	3.32E-10	5.31E-07
UP_SEQ_FEATURE	zinc finger region:C2H2-type 9	28	7.63	4.30	4.58E-10	7.33E-07
UP_SEQ_FEATURE	zinc finger region:C2H2-type 12	23	6.27	5.27	5.15E-10	8.24E-07
SMART	SM00349:KRAB	26	7.08	4.36	7.67E-10	8.65E-07
UP_SEQ_FEATURE	zinc finger region:C2H2-type 15	17	4.63	7.40	1.17E-09	1.88E-06
UP_SEQ_FEATURE	zinc finger region:C2H2-type 7	30	8.17	3.84	1.33E-09	2.13E-06
INTERPRO	IPR001909:Krueppel-associated box	26	7.08	4.20	3.15E-09	4.49E-06
UP_SEQ_FEATURE	domain:KRAB	25	6.81	4.37	3.38E-09	5.41E-06
UP_SEQ_FEATURE	zinc finger region:C2H2-type 14	17	4.63	6.32	1.19E-08	1.90E-05
UP_SEQ_FEATURE	zinc finger region:C2H2-type 13	19	5.18	5.50	1.19E-08	1.91E-05
UP_SEQ_FEATURE	zinc finger region:C2H2-type 8	27	7.36	3.73	1.86E-08	2.98E-05
UP_SEQ_FEATURE	zinc finger region:C2H2-type 6	29	7.90	3.42	3.22E-08	5.15E-05
INTERPRO	IPR001089:Small chemokine, C-X-C	7	1.91	29.85	4.94E-08	7.06E-05
INTERPRO	IPR002473:Small chemokine, C-X-C/Interleukin 8	7	1.91	27.72	8.52E-08	1.22E-04
GOTERM_BP_FAT	GO:0050877 neurological system process	48	13.08	2.21	2.61E-07	4.27E-04
INTERPRO	IPR018048:Small chemokine, C-X-C, conserved site	7	1.91	22.83	3.35E-07	4.79E-04
INTERPRO	IPR002337:Haemoglobin, beta	5	1.36	55.44	5.04E-07	7.20E-04
INTERPRO	IPR013087:Zinc finger, C2H2-type/integrase, DNA-binding	30	8.17	2.77	1.34E-06	0.002
SMART	SM00355:ZnF_C2H2	33	8.99	2.48	1.77E-06	0.002
SP_PIR_KEYWORDS	receptor	52	14.17	2.00	2.39E-06	0.003
UP_SEQ_FEATURE	zinc finger region:C2H2-type 5	27	7.36	2.90	2.39E-06	0.004
UP_SEQ_FEATURE	zinc finger region:C2H2-type 3	29	7.90	2.70	3.79E-06	0.006
GOTERM_MF_FAT	GO:0047760 butyrate-CoA ligase activity	5	1.36	38.47	3.81E-06	0.005
INTERPRO	IPR007087:Zinc finger, C2H2-type	33	8.99	2.43	5.58E-06	0.008
PIR_SUPERFAMILY	PIRSF002522:CXC chemokine	6	1.63	20.55	6.13E-06	0.007
SP_PIR_KEYWORDS	oxygen carrier	5	1.36	35.19	6.39E-06	0.009
INTERPRO	IPR015880:Zinc finger, C2H2-like	33	8.99	2.39	7.71E-06	0.011
GOTERM_BP_FAT	GO:0007166 cell surface receptor linked signal transduction	59	16.08	1.78	9.41E-06	0.015
UP_SEQ_FEATURE	zinc finger region:C2H2-type 16	11	3.00	6.18	1.14E-05	0.018
PIR_SUPERFAMILY	PIRSF500045:hemoglobin, vertebrate type	5	1.36	29.97	1.16E-05	0.013
UP_SEQ_FEATURE	zinc finger region:C2H2-type 17	10	2.72	7.02	1.20E-05	0.019
UP_SEQ_FEATURE	disulfide bond	77	20.98	1.62	1.27E-05	0.020
UP_SEQ_FEATURE	topological domain:Extracellular	75	20.44	1.62	1.99E-05	0.032
PIR_SUPERFAMILY	PIRSF005559:zinc finger protein ZFP-36	13	3.54	4.58	2.22E-05	0.025
SP_PIR_KEYWORDS	disulfide bond	78	21.25	1.59	2.44E-05	0.033
UP_SEQ_FEATURE	zinc finger region:C2H2-type 20	7	1.91	11.56	2.64E-05	0.042
SP_PIR_KEYWORDS	blood	5	1.36	25.59	2.89E-05	0.039
SP_PIR_KEYWORDS	cell membrane	63	17.17	1.70	3.07E-05	0.041

References

Huang BDW and Lempicki R. 2008. Systematic and integrative analysis of large gene lists using DAVID bioinformatics resources. *Nature protocols* pp. 1–43.

Huang DW, Sherman BT, and Lempicki Ra. 2009. Bioinformatics enrichment tools: paths toward the comprehensive functional analysis of large gene lists. *Nucleic acids research* **37**: 1–13.
